# Genome-Wide Expression Analysis of Soybean MADS Genes Showing Potential Function in the Seed Development

**DOI:** 10.1371/journal.pone.0062288

**Published:** 2013-04-30

**Authors:** Cheng-Ming Fan, Xu Wang, Yan-Wei Wang, Rui-Bo Hu, Xiao-Mei Zhang, Jian-Xin Chen, Yong-Fu Fu

**Affiliations:** 1 MOA Key Lab of Soybean Biology (Beijing), National Key Facility of Crop Gene Resource and Genetic Improvement, Institute of Crop Sciences, Chinese Academy of Agricultural Sciences, Beijing, China; 2 College of Life Sciences, Henan Agricultural University, Zhengzhou, Henan, China; 3 CAS Key Laboratory of Biofuels, Shandong Provincial Key Laboratory of Energy Genetics, Qingdao Institute of BioEnergy and BioProcess Technology, Chinese Academy of Sciences, Qingdao, Shandong, China; Nanjing Agricultural University, China

## Abstract

The MADS family is an ancient and best-studied transcription factor and plays fundamental roles in almost every developmental process in plants. In the plant evolutionary history, the whole genome duplication (WGD) events are important not only to the plant species evolution, but to expansion of members of the gene families. Soybean as a model legume crop has experience three rounds of WGD events. Members of some MIKC^C^ subfamilies, such as SOC, AGL6, SQUA, SVP, AGL17 and DEF/GLO, were expanded after soybean three rounds of WGD events. And some MIKC^C^ subfamilies, MIKC* and type I MADS families had experienced faster birth-and-death evolution and their traces before the *Glycine* WGD event were not found. Transposed duplication played important roles in tandem arrangements among the members of different subfamilies. According to the expression profiles of type I and MIKC paralog pair genes, the fates of MIKC paralog gene pairs were subfunctionalization, and the fates of type I MADS paralog gene pairs were nonfunctionalization. 137 out of 163 *MADS* genes were close to 186 loci within 2 Mb genomic regions associated with seed-relative QTLs, among which 115 genes expressed during the seed development. Although MIKC^C^ genes kept the important and conserved functions of the flower development, most MIKC^C^ genes showed potentially essential roles in the seed development as well as the type I MADS.

## Introduction

The MADS family, found in fungi [1], animals [2] and plants [3] [4], possesses a highly conserved N-terminal with a DNA-binding domain named MADS. Based on the phylogenetic analysis, MADS gene family is divided into two large lineages, type I and type II, which was created through a gene duplication occurred before the divergence of plants (and fungi) and animals [5]–[7].

In plant, the typical difference between type II MADS genes and type I MADS genes is that the plant type II, but not type I, has a K-domain [5], [6], [8]. The plant-special type II MADS is also named as **MIKC** MADS due to their four domains, **M**ADS domain, **I** domain, **K** domain, and **C** domain [8], [9]. and except MADS-domain and K-box domain, I-domain and C-domain are not conserved although they have important functions for the MADS family [9], [10]. MIKC type can be further divided into MIKC^C^ and MIKC* clade, both of which were present in a common ancestor of mosses and vascular plants, suggesting they are an ancestral kind of genes [11]. About 13 subfamilies compose of the MIKC^C^ clade and most of them originate from ancestral seed plants and are often characterized by distinct sequence motifs in their C-terminal domains [5], [12]. And based on the phylogenic tree, AG-, AGL6-, AGL12-, DEF+GLO- (B), GGM13- (B(s)), STMADS11- and TM3-like genes very likely existed already in the most recent common ancestor of angiosperms and gymnosperms and AGL2-, AGL17-, and SQUA-like genes, existed at least already in the most recent common ancestor of monocots and eudicots [5]. MIKC* clade is characterized by an altered protein domain structure, probably evolved from an ancestral MIKC^C^ gene by a duplication in the Keratin-like region and composed of S-clade, P-clade, lycophyte-clade and Bryophyte-clade [7], [13]. Heterogeneous type I MADS can be subgrouped into Mα, Mβ, and Mγ based on the sequence of the MADS domain and the presence of additional motifs [5], [14]–[16].

Since the plant MADS genes, *AGAMOUS* (*AG*) from *Arabidopsis thaliana* [3] and *DEFICIENS* (*DEF*) from *Antirrhinum majus* [4] are first discovered as regulators of floral organ identity, a lot of plant MIKC^C^ genes have been isolated from various plant species and demonstrate their essential roles in almost all developmental processes in plants, such as the control of flower identity, root architecture, gametophyte development, fruit ripening, the regulation of flowering time ([17]–[20]). By contrast, less attention is paid for type I MADS genes. Recent studies indicate a key regulatory role for type I MADS genes in plant specifying female gametophyte, embryo, and endosperm development [20], [21]. MIKC* genes retained a conserved role in the gametophyte during land plant evolution [13]. And five *Arabidopsis* MIKC* genes (*AGL30*, *AGL65*, *AGL66*, *AGL94*, and *AGL104*) are expressed in pollen and regulate pollen development by repressing immature pollen genes and activating mature pollen genes [22], [23].

Extensive duplications in the angiosperms have resulted in the expansion of members of the gene families and gene diversifications. And duplications in plant MADS transcription factors have been studied to understand the origins and evolution of plant developmental mechanisms [24], [25], and the results demonstrated that duplicated genes either swapped roles, acquired novel roles or retained ancestral roles in different plant species [26]. The modern soybean genome has apparently undergone one whole-genome triplication (WGT) and two whole genome duplication (WGD) events (Legume WGD and Glycine WGD), and about 75% of genes have multiple paralogs [27], [28]. Among them, ∼50% of paralogous genes display expression subfunctionalization [29], which may contribute to phenotypic variation in polyploids [30]. In addition to WGD duplication, tandem duplication generates gene copies that are consecutive in the genome and is presumed to arise through unequal chromosomal crossing over [31] and may contribute to the expansion of some large gene families [32]. Dispersed duplicates are neither adjacent to each other in the genome nor within homeologous chromosome segments and may result from transposition events [31], [33]. Such distantly transposed duplications may occur by DNA-based or RNA-based mechanisms [34]. A total of 32,552 retrotransposons (Class I) and 6,029 DNA transposons (Class II) are found in the soybean genome [35]. And transposed duplication may play significant roles in shaping and reshaping of their host genomes regulating gene expression, altering gene function, and creating new genes [36], [37].

MADS families are identified and classified in many flowering plants such as *Arabidopsis* [14], petunia [38], tomato [39], poplar [40], rice [41], grapevine [42], maize and sorghum [43], cucumber [44]. But only genome-wide expression profiles of all the MADS genes have been reported in *Arabidopsis* and rice [14], [15], [41], and a comprehensive plant protein-protein interactome map of nearly all members of the *Arabidopsis* MADS family has been constructed to investigate the essential roles of Arabidopsis biological processes [45]. Expression profiles of MIKC^C^ genes of conserved known biological functions are systematically analyzed in some plants [39], [42].These accomplishments benefit us to comprehensively understand the plant MADS family and their functions in plant development.

Soybean is one of the most important crop plants for seed protein and oil content, which are associated with the seed development, and genetic control of agronomic traits, such as seed constituents and yield, is inherited in a quantitative manner. Based on the soybase database (http://www.soybase.org) [46], about 2200 soybean quantitative trait loci (QTLs) were associated with about 134 soybean agronomic traits, among which about 1000 QTLs were relative to the seed or pod development. Furthermore, the soybean genome has been completely sequenced [47]. Therefore, QTLs can be mapped to the genome and provide the primary insight for understanding potential functions of co-localized genes in the soybean development.

The MADS family has obviously functional roles in the plant development. Soybean MADS genes and the characters of their evolution in the soybean genome evolution process were identified through the bioinformatic analysis. And based on the genome-wide expression patterns through *in silico* expressions and RT-qPCR, more attentions were paid to soybean MADS expressions in the seed development. In addition, co-localizations of MADS genes with QTLs relative to seed traits were investigated. According to our results, not only did type I MADS highly express in the seed development [21], [48], [49], but also MIKC^C^ genes had important functions in the seed development, except the conserved and best-studied function of the flower development.

## Results

### Identification, Motif, Chromosome Location and Gene Structure of *GmMADS* Genes

In total, 163 MADS genes were obtained from soybean genome (*G.max* v1.0) through HMMER v3.0 based on the HMM model of SRF-type transcription factor (PF00319) and named as *GmMADS1* through *GmMADS163* (Table S1). These MADS genes can be phylogenetically classed into several subgroups as *Arabidopsis* MADS does [5] (Figure S1). These subgroups were type II (MIKC^C^ (81 gnes) and MIKC* (7 genes)) and type I (Mα (36 genes), Mβ (14 genes) and Mγ (24 genes)). And the soybean MIKC^C^ genes contained the MADS domains and K-box domains and were composed of 12 subfamilies: FLC (2 genes), SOC (TM3-like, 8 genes), AG (10 genes), AGL6 (6 genes), SEP (AGL2-like, 12 genes), SVP (STMADS11-like, 8 genes), AGL12 (2 genes), AGL15 (2 genes), AGL17 (8 genes), DEF/GLO (11 genes), SQUA (10 genes), and ABS (GGM13- (B(s))-like, 2 genes), and one ancient clade known in some flowering plant species, TM8 subfamily [5], was not found in the soybean. MIKC* has 2 subfamilies, MIKC*-S (4 genes) and –P (3 genes) according to Kwantes, *et al*. [13].

**Figure 1:**
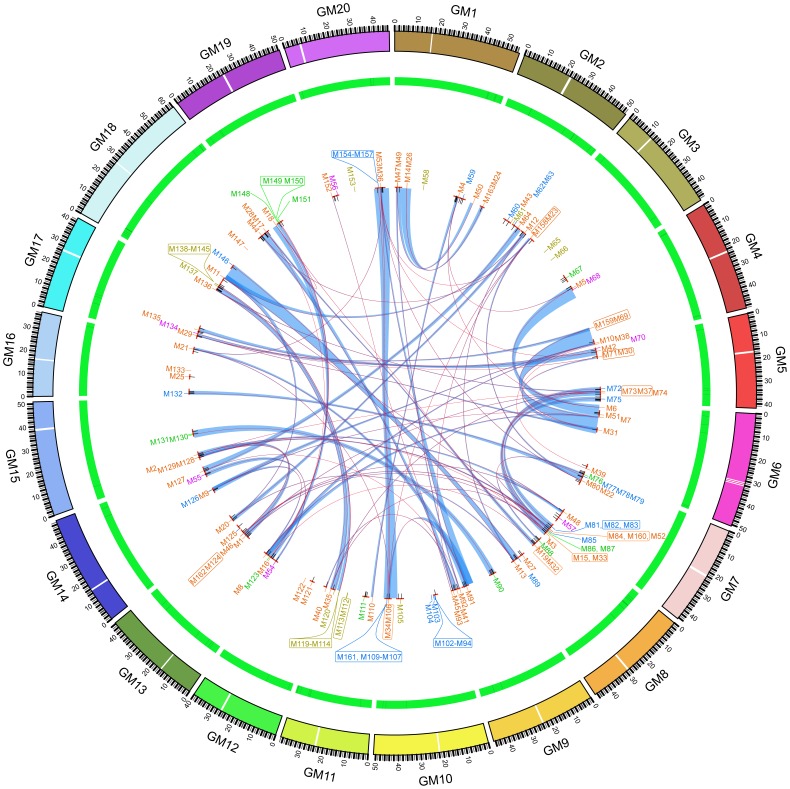
The soybean MADS gene family. The gene names of MIKC^c^, MIKC*, Mα, Mβ and Mγ were abbreviated as M1 to M163 and were in orange, purple, blue, green and yellow, respectively, and short lines in corresponding color in the red blocks showed their locations in the soybean genome (Table S1). The short black lines in the green arcs showed the markers associated with non-seed QTLs and those in the red short blocks showed the markers associated with seed QTL (Table S4). The red blocks showed regions of QTLs relative to the seed traits according to the markers (Table S4). The light blue rainbows showed collinear relationships among the blocks containing *MADS* genes according to the MCScanX results (Table S3) and the red curves showed the paralogs. Twenty chromosomes (GM1-20) were in different colors and the size of the arc showed the size of chromosome (Mb). The figure was created through the software Circos (http://circos.ca/).

*GmMADS* genes were distributed on 20 chromosomes, especially in GM8 (18 MADS genes), GM10 (16 MADS genes) and GM18 (18 MADS genes) (Figure 1). And according to the characters of MADS gene distributions, the location of MIKC and Mγ genes were not biased in the chromosomes, but Mα genes mainly located in the end of GM10 through tandem or proximal duplicates, as well as Mβ genes in GM11 and GM18.

**Figure 2:**
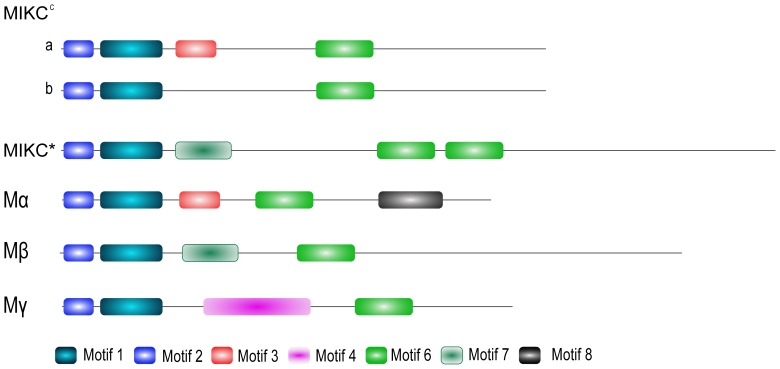
Schematic diagrams of motif organizations. MIKC^C^ can be grouped into two type: a and b (Figure S2). The average length of members of each five families was as the length of a family. Motif 1 and 2 are equivalent to the MADS-box domain (PF00319), and motif 3 and motif 6 is the part of the I-domain and K-box domain (PF01486) for type II MADS proteins, respectively. Other motifs was unknown. The detailed information of soybean MADS motif organizations referred to Figure S2.

Based on the motif organization of 163 soybean MADS proteins, all soybean MADS proteins almost had three motifs, motif 1, 2 and 6 (Figure 2 and Figure S2). And motif 1 (29 aa, Table S2) and 2 (14 aa, Table S2) is localized in the MADS-box domain, while motif 6 (27 aa, Table S2) in the K-box domain. That indicated motif 1, 2 and 6 were very important to the function of the MADS family. Besides motif 1, 2 and 6, some subfamilies of MIKC^C^, MIKC*, Mα, Mβ and Mγ had their own typical motifs (Figure 2). Some members of subfamilies of MIKC^C^, such as AG (GmMADS1, 2, 3, 11, 13, 20, 39 and 74), SOC (GmMADS44, 45, 53, 80, 106, 135 and 158), SVP (GmMADS48-52, 124, 128 and 162), AGL17 (GmMADS46, 64, 92, 129 and 160), AGL6 (GmMADS22, 23 and 91), AGL15 (GmMADS43) and SQUA (GmMADS32), contained motif 3, which was localized in I-domain. In addition, most Mα members contained motif 3 and motif 8. Members of the MIKC* and Mβ family shared motif 7. Furthermore, MIKC* proteins had the double motif 6, whereas members of the Mγ family had motif 4.

In term of the gene structure, the fist exon (about 180 bp) conservatively coded the MADS domain in the MADS genes with more than one exons (Table S1). 7 MIKC* genes had 9–11 exons, and covered about 4.7 kb in length in the genome. For the MIKC^C^ genes, the number of exons were 4–9, and average number was about 7, and covered about average about 7.4 kb in length from 0.75 kb to 18.6 kb, and genome sequences of about 82.7% MIKC^C^ genes were longer than 5 kb. About 44/75 type I MADS genes had only one exon and 12/75 type I MADS genes had more than 4 exons, and the average exon number of Mα, Mβ, and Mγ genes was 1.6, 2.4, and 2.1 respectively, and the average genome sequences were about 0.6, 1.2, and 0.8 kb respectively.

### Different Expansion Patterns of Two Types of MADS within the Soybean Genome

To investigate *GmMADS* gene evolution in soybean, the syntenic relationships among *G.max*, *M.truncatula*, experiencing the WGT and Legume WGD events [50] and *V.vinifera*, experiencing the WGT event [51], were computed through *MCScanX* [52]. Based on our results, the blocks containing some MIKC genes, which belonged to SOC, AG, SQUA, AGL6, SEP, SVP, DEF/GLO and MIKC*, can be found the corresponding homologous blocks in *M.truncatula* and/or *V.vinifera* genome (Table S3). But for the type I genes, the inter-species syntenic relationships can hardly been found. That indicated gene orders of the blocks containing MIKC genes were more conserved than that containing type I MADS genes during the soybean evolution process.

**Figure 3:**
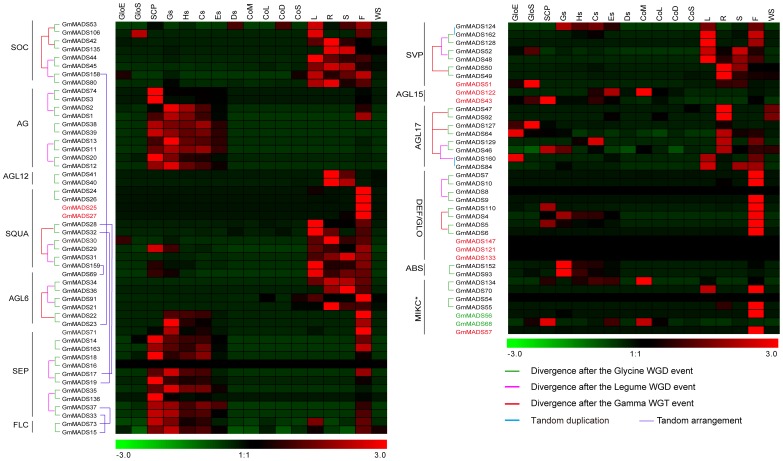
*In silico* expression profiles and the evolutional pattern of soybean MIKC genes. The RNA-seq relative expression data of 17 tissues was used to re-construct expression patterns of *MIKC* genes. 3 samples from soybean seed compartments: GloE (Globular stage embryo proper), SCP (Early maturation seed coat parenchyma) and GloS (Globular stage suspensor); 10 soybean tissues samples: Gs (Globular Stage Seed), Hs (Heart Stage Seed), Cs (Cotyledon Stage Seed), Es (Early Maturation Stage Seed), Ds (Dry Seed), R (Root), S (Stem), L (Trifoliate leave), F (Floral bud), and WS (Whole seedling six days after imbibition); 4 soybean cotyledon development samples: CoM (Mid-maturation cotyledon), CoL (Late-maturation cotyledon), CoD (Dry seed) and CoS (Seedling cotyledon). The raw data was downloaded from the website http://seedgenenetwork.net/presentation. Gene names in red showed dispersed duplicate, in blue showed proximal duplicate, and in green its paralog genes were lost during evolution. The lines showed the blocks containing the corresponding MADS genes experienced the WGD events, and the evolution models of the blocks were displayed in Figure 1 and S5. The raw relative expressions of 163 MADS genes were in the Table S6.

WGD events expanded the members of the MIKC family, and blocks containing about 85% (75/88) MIKC genes experienced WGD events (Figure 1, 3 and Table S3). Based on syntenic blocks, all the genes of subfamily AG (10 genes), AGL12 (2 genes), ABS (2 genes), FLC (2 genes), and SOC (8 genes) were originated from 3, 1, 1, 1, and 1 different ancestor sites before the Gamma WGT events respectively. In addition to the WGD duplication, other gene duplication patterns were found in some MIKC^C^ subfamilies. In *SQUA* subfamily, six paralog genes (*GmMADS28/32*, *29/30*, and *31/159*) were the resultants of a common ancestor sites experiencing three WGD events, and one paralog gene pair (*GmMADS24/26*) were diverged after the *Glycine* WGD event, while *GmMADS25* and *GmMADS27* were the results of the transposed duplication (Table S3–4). Seven members of *AGL17* subfamily were originated from an ancestor sites before the Gamma WGT events, and *GmMADS84* was tandem connection to *GmMADS160*. In SVP subfamily, six paralog genes were derivates of a common ancestor, while *GmMADS124* and *162* through the tandem duplication and a dispersed gene (*GmMADS51*) through the transposed duplication (Table S3–4). Eight DEF/GLO genes were originated from two different ancestor sites, and the transposable elements occurred at the up/dwonstrean of *GmMADS121*, *133*, and *147*, which were dispersed in the genome (Table S-3). The origins of 10/11 SEP genes were 3 different ancestor sites before the Gamma WGT events, and a transposed duplication member, *GmMADS71*, was proximal to *GmMADS23* (AGL6) (Table S3–4). Three paralog gene pairs of the AGL6 subfamily *GmMADS21*/*91*, *GmMADS22*/*23* and *GmMADS34*/*36* were from the common ancestor site before three rounds of WGD events, but *GmMADS69* located among two transposable elements had the tandem relationship with *GmMADS159* (SQUA) (Table S3–4).

Only AGL15 subfamily had not any paralog genes and was composed of two dispersed genes, *GmMADS122* (GM12) and *GmMADS43* (GM2), which were located among two transposable elements (Table S4). In the syntenic blocks, some MIKC^C^ genes of different families showed the tandem relationship. For example, paralog SEP gene pairs *GmMADS33*/*37* and *GmMADS17*/*19* displayed tandem relationship with *GmMADS15*/*73* (FLC subfamily) and *GmMADS28*/*32* (SQUA subfamily) respectively (Figure 1). That may result from the transposable duplications (Table S4).

For MIKC* gene family, the members of MIKC*-S (*GmMADS54*/*55* and *GmMADS68*) and –P (*GmMADS70*/*134* and *GmMADS56*) experienced the same evolution processes. The paralog pair genes were diverged after the *Glycine* WGD event (Figure 1, 3 and Table S3). And *GmMADS56* and *GmMADS68* did not have the corresponding gene pairs in the soybean syntenic blocks, but their homologous blocks and collinear pairs were found in *Medicago* (two MIKC*genes, *MtMADS52* and *MtMADS23* respectively) (Table S3), suggesting that their collinear genes in their soybean syntenic blocks were not retentive after the Legume WGD events.

**Figure 4:**
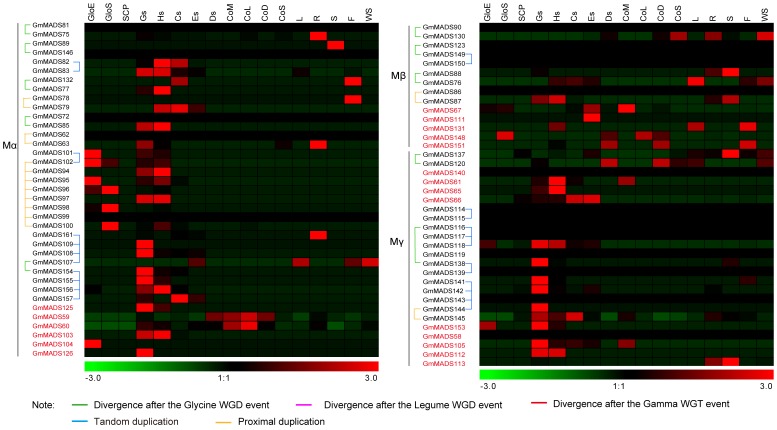
*In silico* expression profiles and the evolutional pattern of soybean type I MADS genes. Notes as Figure 3.

Multiple duplications made the number of the type I *GmMADS* expansion. 5 Mα, 3 Mβ and 2 Mγ paralog gene pairs were diverged only after the *Glycine* WGD event (Figure 1, 4 and Table S3). And 10 Mα, 1 Mβ and 8 Mγ genes were generated through tandem duplications, and 11 Mα, 2 Mβ and 1 Mγ genes were generated through proximal duplications, and 6 Mα, 9 Mβ and 15 Mγ genes were dispersed through the transposed duplication in the genome (Figure 1, 4 and Table S4). That suggested that type I genes had experienced a higher rate of birth-and-death evolution than type II genes.

### Soybean MADS Genes Co-localized with QTLs for the Seed-relative Features

Based on the soybean QTL database (http://www.soybase.org/search/qtllist.php), about 269 loci associated with 807 QTLs for 112 traits were found within 2-Mb genomic regions surrounding 148 soybean MADS genes (Figure 1 and Table S4). And 186 out of 269 loci were associated with 372 QTLs for 59 seed-relative traits, containing 295 QTLs relative to seed traits (constituent or size), 32 to pod maturity date, one to R3 beginning pod, one to R8 full maturity and 43 to the yield (Table S5). And 137 MADS genes (all members of Mα, Mβ and MIKC* family and 67 MIKC^C^ genes and 12 Mγ genes) localized in 2-Mb genomic regions near to 186 loci associated with the seed-relative QTLs (Figure 1 and Table S5).

In addition, according to the *in silico* transcriptome (Figure 3, 4 and Table S6), transcriptions of 129 soybean MADS genes were detected in the seed tissues. However, 11/129 genes (*GmMADS14*, *26*, *50*, *65*, *66*, *69*, *112*, *113*, *122*, *153* and *159*) were not co-localized with any QTLs, and 3/129 genes (*GmMADS6*, *18* and *110*) were not with QTLs relative to the seed traits (Table S5). That indicated 115 MADS genes may be involved in the seed development.

### Soybean MADS Genes Showing Highly Expression in the Seed Development through *in silico* Transcriptome

For the whole expression analyses of the soybean MADS family, the RPKM method was employed to correct biases in total gene exon size and to normalize for the total short read sequences obtained in 17 tissue libraries [53], [54]. And then relative RPKM values represented the relative expression of each MADS genes (Figure 3, 4 and Table S6). From the online database, 25 *GmMADS* genes (19 for type I and 6 for type II), were undetectable at the transcription level in all 17 tissues; 29 genes (22 for Mα, 1 for Mβ and 6 for Mγ, were detected only in seed tissues; 9 genes, (5 for MIKC^c^, 2 for MIKC*, and 2 for Mα, were detected only in non-seed tissues; 37 genes (34 for MIKC and 4 for type I), had no biased tissues and wide expression with fluctuant levels.

**Figure 5:**
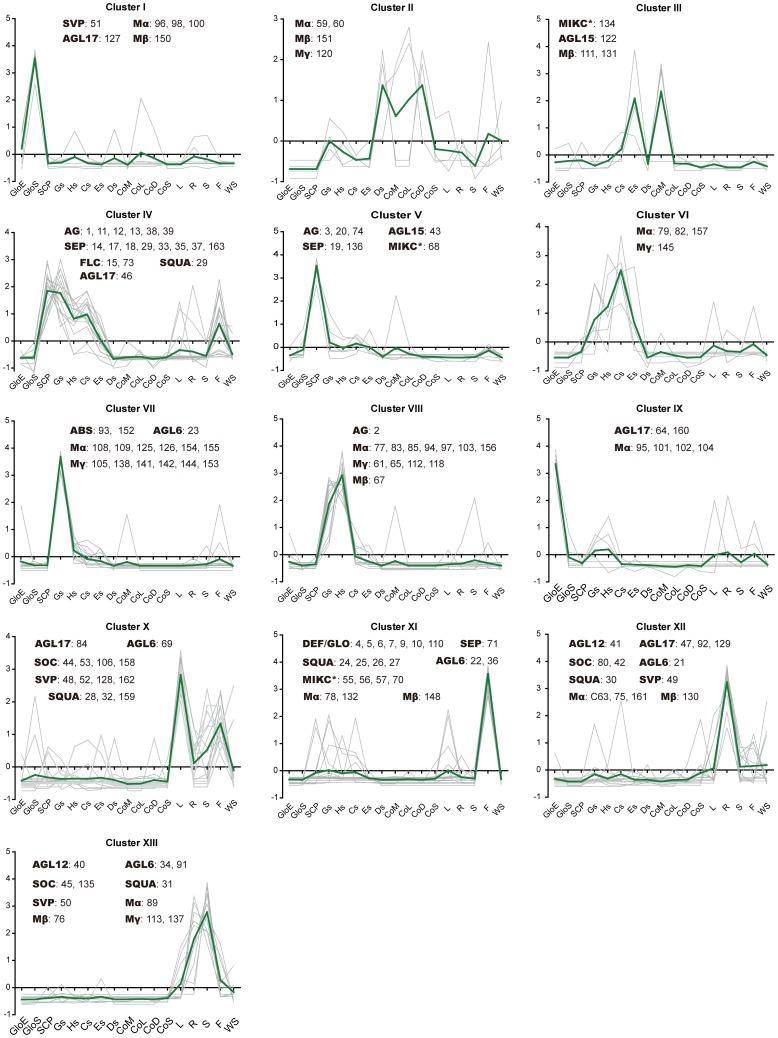
Expression cluster analysis based on *in silico* expression of 138 MADS genes. The samples were designed as Figure 3. The gray line shows expression profiles of each genes, and the green line is the average expression and indicates the expression pattern of one cluster. For simplicity, gene names display the corresponding code numbers of every subfamily (Figure S1 and Table S6).

A hierarchical clustering analysis of transcription profiles in 17 tissues based on a Pearson correlation displayed 13 clusters for 138 *GmMADS* genes (Figure 5 and S3). Most of them (78 genes, 43 for type I and 35 for *MIKC*) highly expressed in the seed tissues and fell into Cluster I to IX. The genes in different clusters had their own abundant transcripts in different tissues, such as Cluster I and IX in suspensors and embryos at globular stage, respectively, both Cluster IV and V in seed coat parenchymas at the early stage of seed maturation, Cluster VII in seeds at the globular embryo stage, Cluster VIII in seeds at both the globular and heart embryo stages, both Cluster IV and Cluster VI at the seed developing stages, Cluster II in developing cotyledons or in dry seeds, and Cluster III in seeds at the early stage of seed maturation.

There were 36 *GmMADS* genes of Cluster X and XI highly expressed in flowers. And Cluster X genes also highly expressed in leaves. Cluster XII expressed mainly in roots, while Cluster XIII in both roots and stems (Figure 5 and S3).

By and large, the expression level of type I *GmMAD*S genes was much lower than that of MIKC, and they were mainly in the seeds as the previous report in other species [49]. The function of MIKC genes is famous as regulators in the floral organ development, but our analysis showed that high expression of some MIKC genes were detected in roots, stems, leaves, and seeds besides flowers in soybean (Figure 5), indicating that soybean MIKC genes had extensive functions in developmental progresses.

### Expression Profiles of *GmMADS* Genes by RT-qPCR

To confirm the expression profiles above, Real-time quantitative PCR (RT-qPCR) was employed to evaluate the transcripts of 96 genes in different tissues at different stages. Transcriptions of 83 MADS genes were consistent with the transcription profiles above (Figure S3 and S4) in most cases, but the expressions of *GmMADS4*, *48*, *52*, *55*, *56*, *57*, *70*, *93*, *96*, *112* and *126* were undetectable in all the 12 samples, and *GmMADS8* and *16* can be detected in the flowers.

### *GmMADS* Gene Expression Peaks in the Soybean Seeds

**Figure 6:**
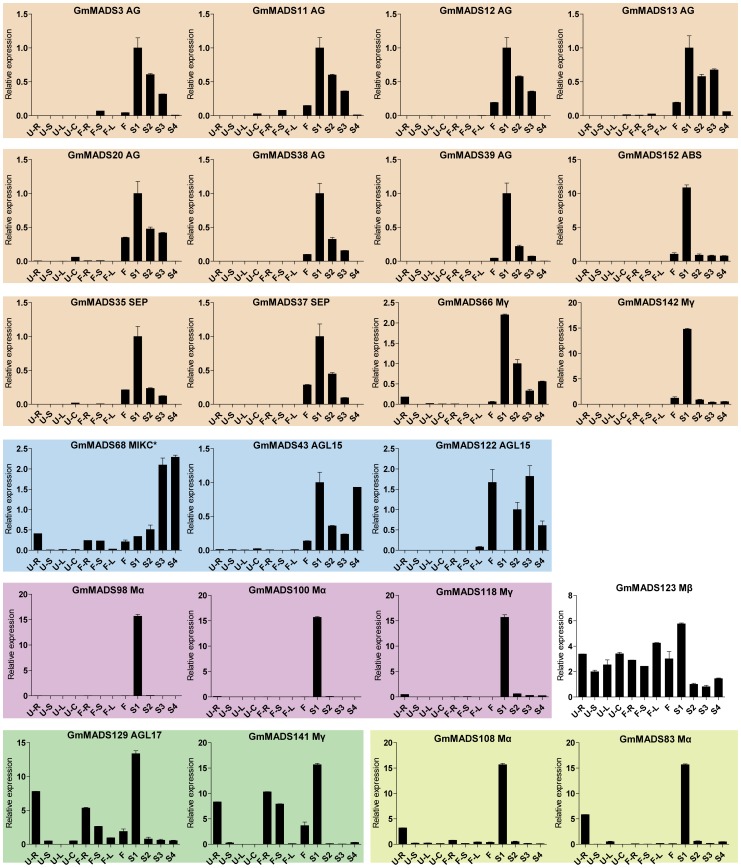
Expression patterns of *GmMADS* genes in the seeds by RT-qPCR. 4 samples at the seedling stage (the unifoliolates open fully): U-R (roots), U-S (stems), U-C (cotyledons) and U-U (leaves); 4 samples at the flowering stage: F-R (roots), F-S (stems), F-L (leaves) and F (flowers); 4 seed development samples: S1 (seeds at 7 days after flowering), S2 (seeds at 14 days after flowering), S3 (seeds at 21 days after flowering) and S4 (dry seeds). The similar expression profiles were in the similar color background. The bar is the average with standard deviation of the expression levels among three different replicates. The geometric means of *GmSKIP16, GmUNKI* and *GmUNKII* transcripts were used as the reference transcript. The values are means of three replicates, and each replicate represented a pool from at least five plants. Error bars represent SD.

Some *GmMADS* genes strongly accumulated in the seed in different seed developmental stages (Figure 6). One expression tendency of them was that the transcription occurred at high level in the seed at the early stage, and then progressively down-regulated along with seed development; their transcripts were detected at very low level in the flowers and hardly in the dry seeds. Such a kind of genes included 7 AG genes (*GmMADS3*, *11*, *12*, *13*, *20*, *38*, and *39*), 2 SEP genes (*GmMADS35* and *37*), one ABS (*GmMADS152*) and 2 Mγ gene (*GmMADS66* and *142*). And 2 Mα genes (*GmMADS98* and *100*) and 1 Mγ (*GmMADS118*) strongly expressed only in the early seeds and barely in the flowers and other tissues. During the seed maturation process, AGL15 (*GmMADS43* and *122*) expressed in relatively high level and the transcriptions of *GmMADS68* (MIKC*-S) increased with the seed maturation. Expression peaks of two Mα genes, *GmMADS83* and *108*, occurred both in seed at the early stage of the seed development and in the root at the seedling stage, and except the roots at the seedling stage, *GmMADS129* (AGL17), and *GmMADS141* (Mγ) strongly expressed in the roots at the flowering stage. The high expressions of *GmMADS123* (Mβ) were not only in the early seeds, but in other tissues (Figure 6).

### *GmMADS* Gene Expression Peaks in the Soybean Flowers

**Figure 7:**
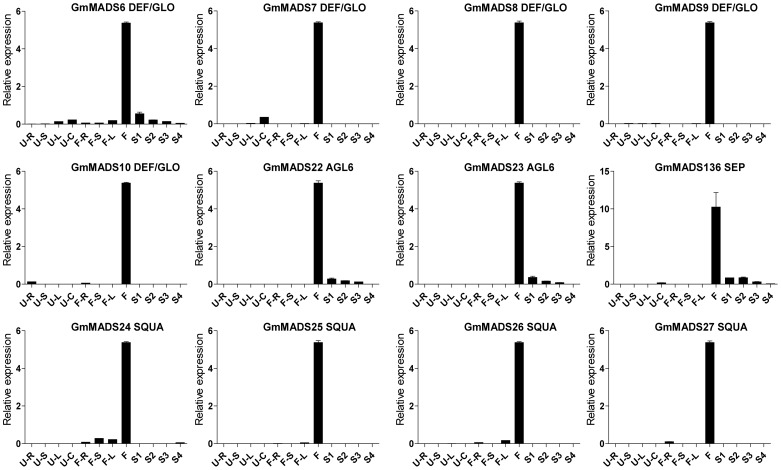
Expression peaks in the flowers through RT-qPCR. Notes as Figure 6.

**Figure 8:**
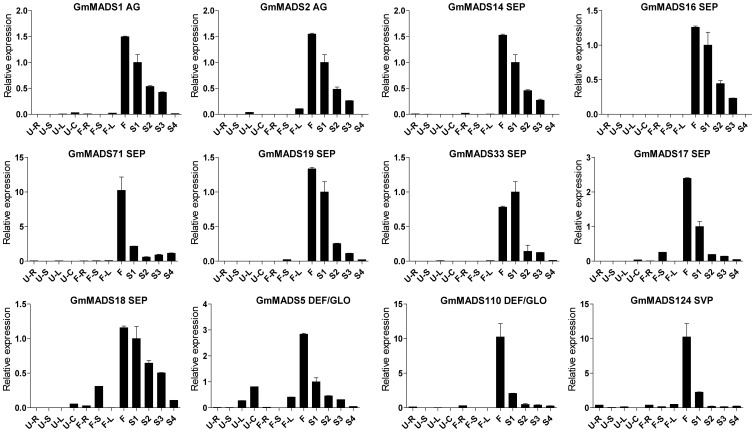
High expression in the seeds and flowers through RT-qPCR. Notes as Figure 6.

**Figure 9:**
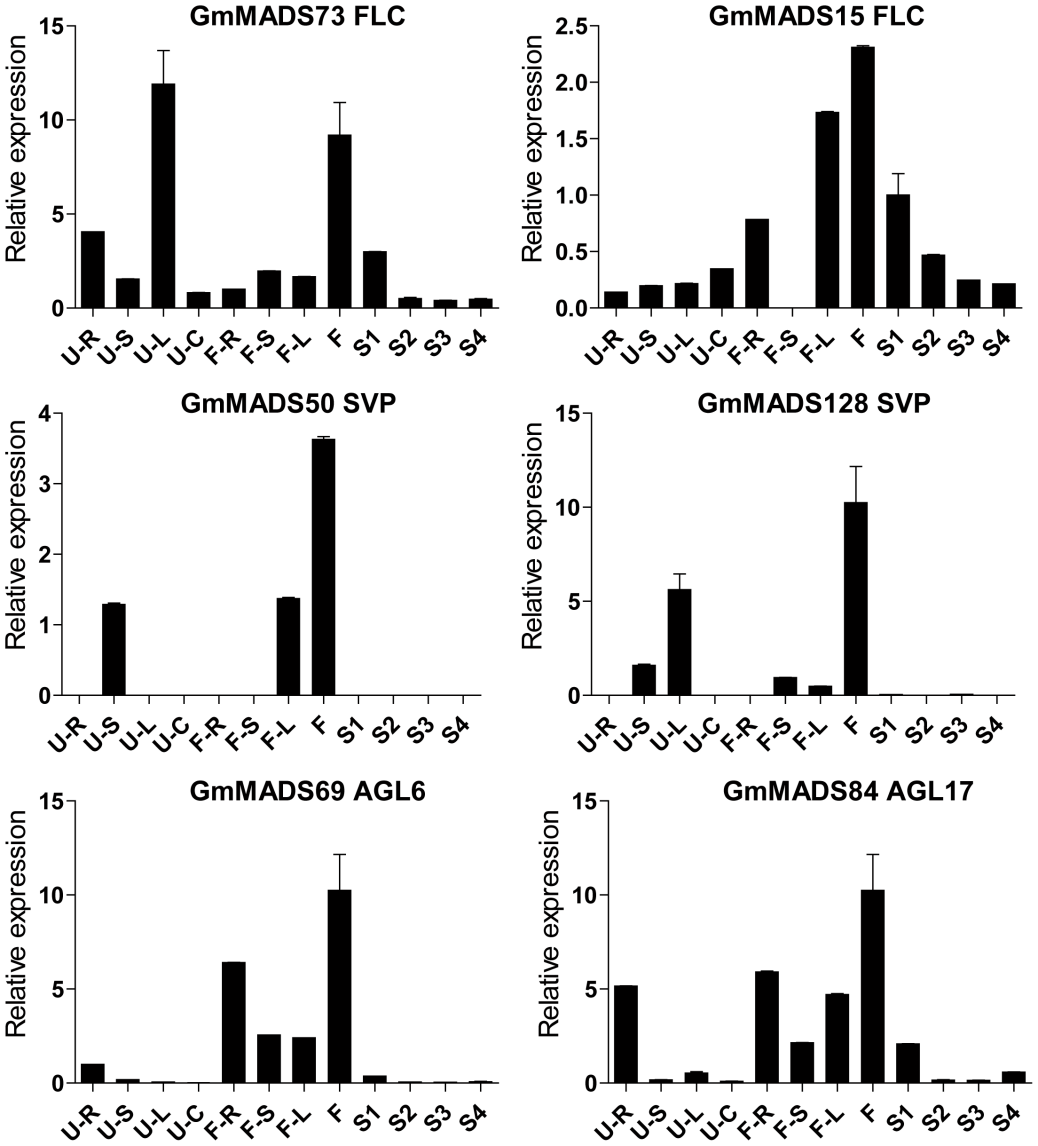
High expression in the flowers and leaves through RT-qPCR. Notes as Figure 6.

*MIKC* genes are well known of the importance in the flower development. In the soybean, transcriptions of MIKC genes were also detectable in the flowers (Figure 7, 8 and 9). For example, DEF/GLO (*GmMADS6*-*10*), SQUA (*GmMADS24*-*27*), AGL6 (*GmMADS2*2 and *23*) and SEP (*GmMADS136*) were most abundant only in flowers (Figure 7). But except in the flowers, SEP genes (*GmMADS14*, *16*, *17*, *18*, *19*, *33*, and *71*), AG genes (*GmMADS1*and *2*), DEF/GLO (*GmMADS5* and *10*), SVP (*GmMADS124*) strongly expressed in the seed development (Figure 8). That indicated these *MIKC^C^* genes played important roles in reproductive tissues.

There were also some genes having relative high abundance in multiple tissues besides the flowers (Figure 9). Two FLC genes (*GmMADS73* and *15*) and two SVP genes (*GmMADS50* and *128*) showed relatively high expression in the leaves at the seedling stage and at the flowering time respectively. The high level of transcripts of *GmMADS69* (AGL6) and *GmMADS84* (AGL17) were in roots, besides in flowers.

### *GmMADS* Gene Expression Peaks in the Soybean Leaves

**Figure 10:**
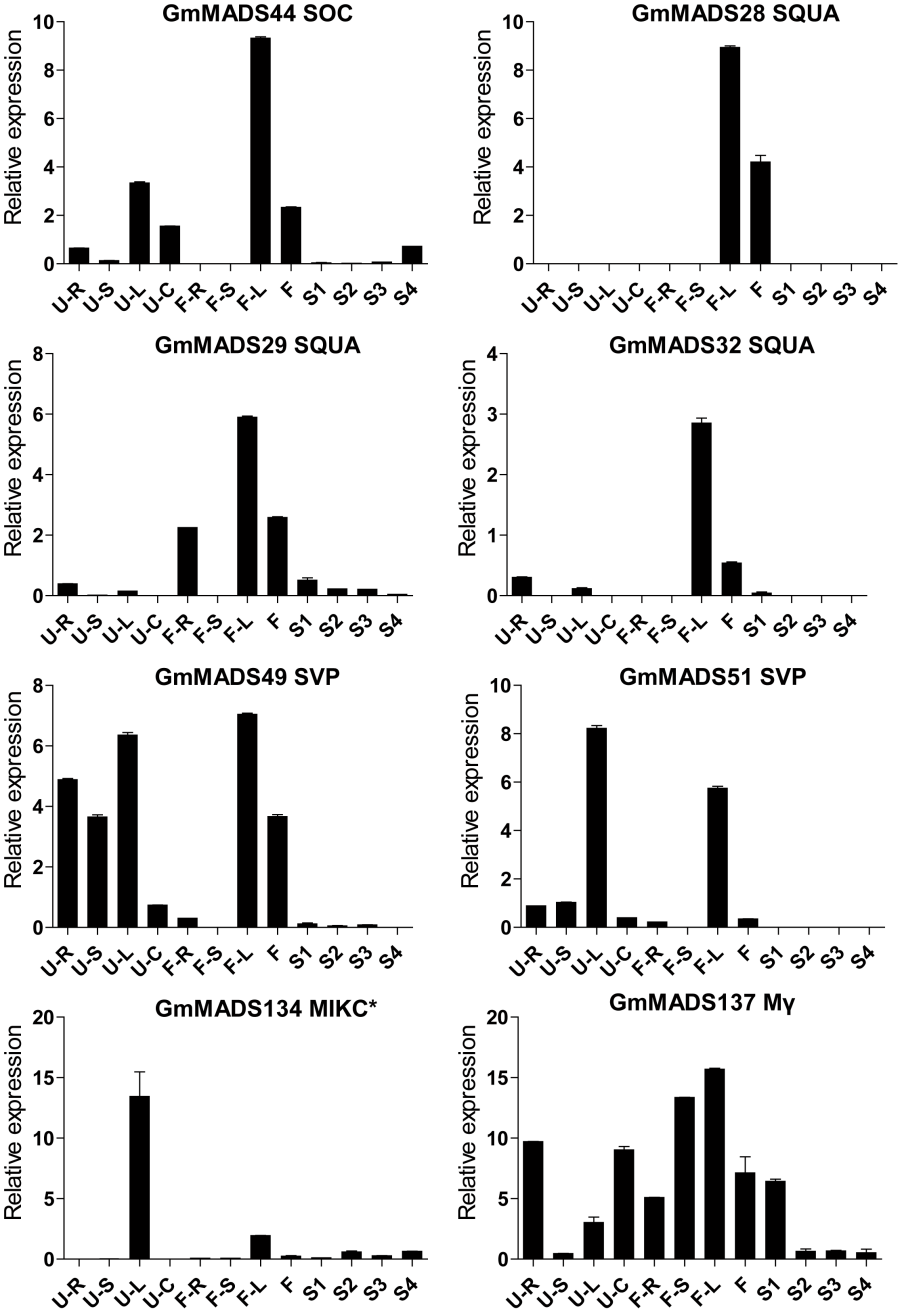
Expression peaks in the leaves through RT-qPCR. Notes as Figure 6.

Expression peaks of 8 *GmMADS* genes occurred mainly in the leaves at the seedling and/or flowering stages (Figure 10). *GmMADS49* and *51* (SVP), *GmMADS44* (SOC) and *GmMADS134* (MIKC*) highly expressed both at the seedling stage and flowering time; *GmMADS28*, *29* and *32* (SQUA) strongly expressed in the leaves and flowers at the flowering time; *GmMADS137* highly expressed not only in the leaves, but in roots, stems and seeds.

### *GmMADS* Gene Expression Peaks in the Soybean Stems

**Figure 11:**
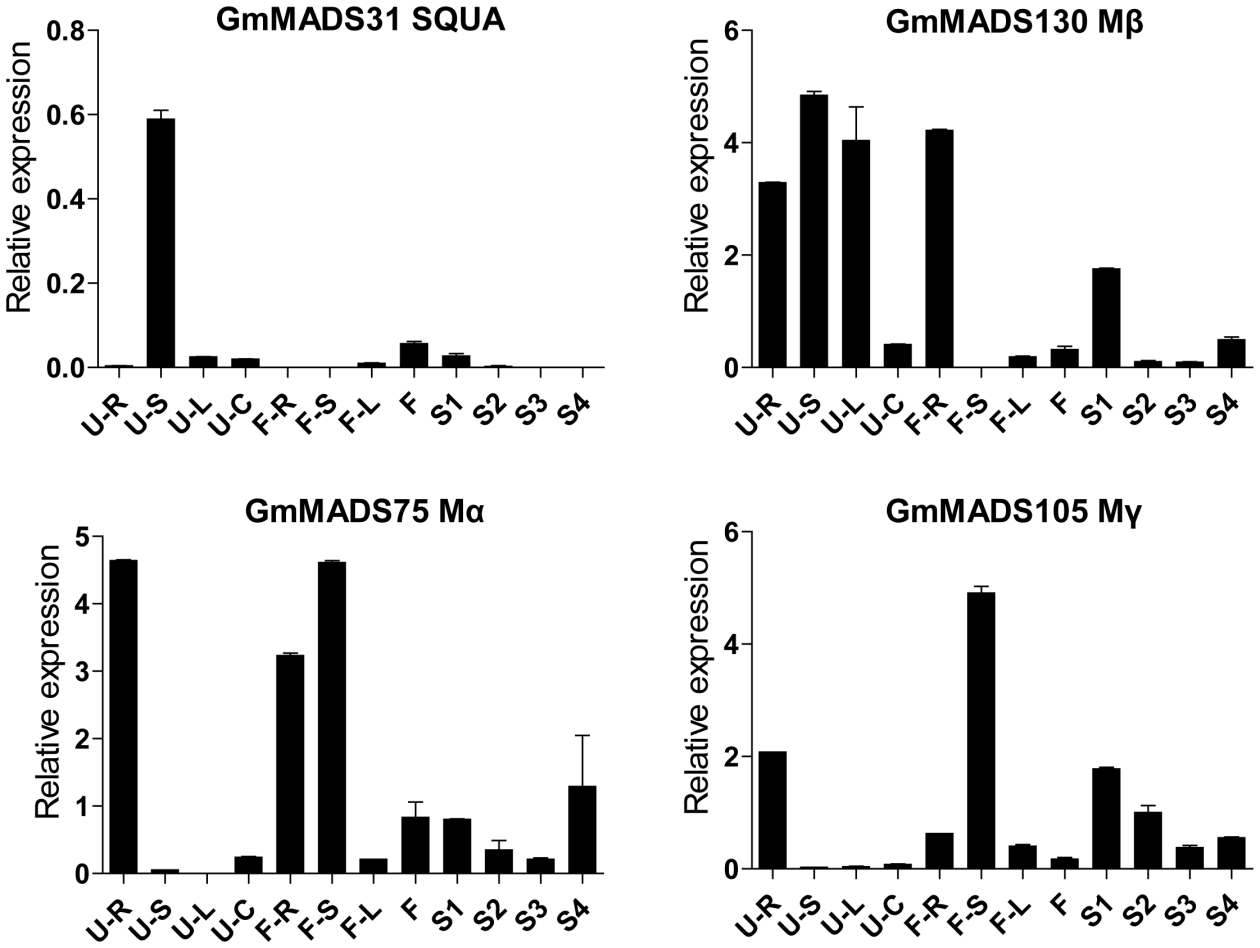
Expression peaks in the stems through RT-qPCR. Notes as Figure 6.

*GmMADS31* (SQUA), *GmMADS75* (Mα), *GmMADS105* (Mγ), and *GmMADS130* (Mβ) highly expressed in the stem (Figure 11). The relative expressions of *GmMADS31* were strongly detected in the seedling stage stems and lower in other tissues. *GmMADS130* highly expressed in the seedling stage stems, but high in the seedling stage roots and leaves and flowering stage roots. The highest transcriptions of *GmMADS75* and *GmMADS105*were in the stems at the flowering time, and they showed relatively high expressions in the roots. And Low transcriptions of all the four genes can also be detected in flower and seeds.

### Expression Peaks of *GmMADS* Genes in Roots

**Figure 12:**
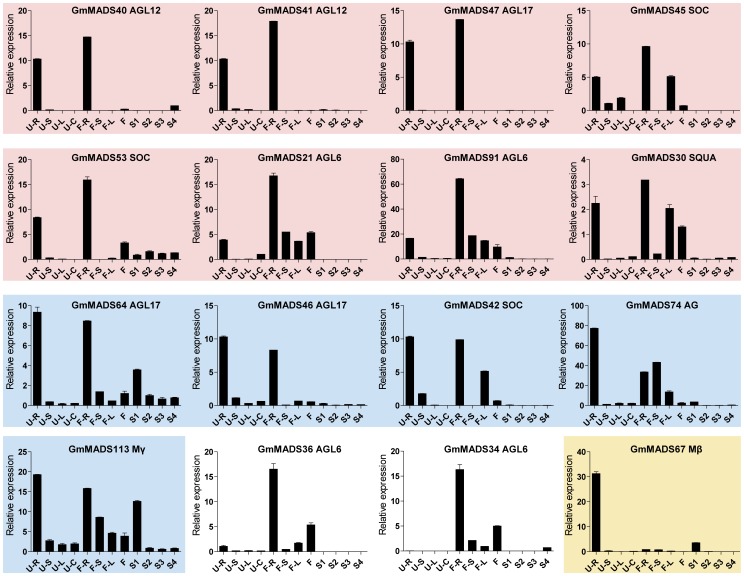
Expression peaks in the roots through RT-qPCR. Notes as Figure 6.

There were about 16 MADS genes, AGL6 (*GmMADS21*, *34*, and *91*), SEP (*GmMADS56*), SOC (*GmMADS42*, *45*, and *GmMADS53*), AGL17 (*GmMADS46*, *47*, and *64*), AGL12 (*GmMADS40* and *41*), SQUA (*GmMADS30*), AG (*GmMADS74*), Mβ (*GmMADS67*), Mγ (*GmMADS113*), showing high levels in roots at vegetative and/or reproductive stages (Figure 12). According to the transcription patterns, eight MIKC^C^ genes expressed more highly in the root at the flowering times than at the seedling stage, and 4 MIKC^C^ gene and one Mγ gene more highly expressed in the seedling stage roots. Expressions of two AGL6 genes were abundant in the roots and other tissues at the flowering time. And *GmMADS67* expressed only in the roots at the seedlings. That indicated 16 genes were more importance of the roots than the flowers and seeds.

### Expression Divergences of the Paralog Gene Pairs

**Figure 13:**
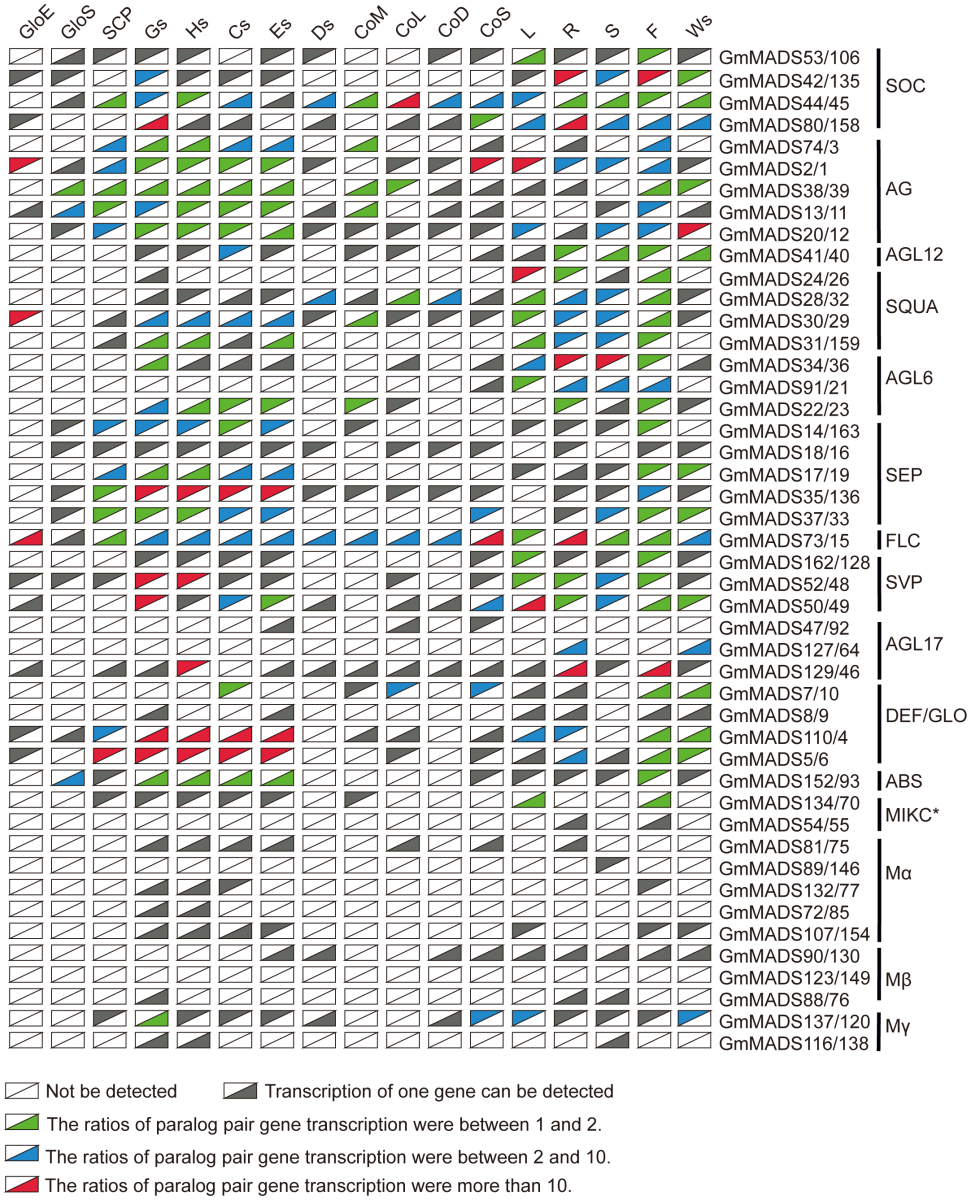
Expression divergence of paralog gene pairs. The upper triangles showed the expression of the lift genes of the paralog gene pairs, and the lower triangle the expression of the right genes of the paralog gene pairs. The raw relative expressions of 163 MADS genes were in the Tab S6. Other notes were similar to Figure 3.

From the results above, there were 46 paralog gene pairs found in the soybean MADS gene family. Based on *in silico* expression data of these pairs in 17 soybean tissues, expression divergence in a sample was obviously evidenced. For example, 236 (about 30.2%) of gene pairs showed only one gene expressed while the other was undetectable; 113 (about 14.5%) of gene pairs showed the ratios between paralog gene pairs were between 1 and 2, 83 (about 10.6%) between 2 and 10, 38 (about 4.9%) above 10 (Figure 13). The MIKC paralog pair gene expressed high or low in same tissues, whereas most one of type I MADS paralog gene pair did not expressed (Figure 13 and Table S3). Our results indicated that, during evolutional progress, MIKC paralog gene pairs underwent sub-funtionalization and type I MADS paralog gene pairs underwent non-functionalization.

## Discussion

### Soybean MADS Subfamilies Experiencing Different Selection Pressure

At least one ancestral MADS-box gene was present in the common ancestor of plants, animals, and fungi, and probably the duplication that gave rise to the animal *MEF2*- and *SRF*-like genes occurred after animals diverged from plants but before fungi diverged from animals about 1000 million year ago (MYA) [6], [8]. Plant *MIKC*-type genes and animal *MEF2*-like genes are monophyletic, not so as plant type I and animal *SRF*-like genes do [6], [8], [16]. Subfamilies of *MIKC*, such as *AG*-, *AGL6*-, *AGL12*-, *DEF*/*GLO*, *GGM13*- (B_(s)_), *STMADS11*- and *TM3*-like genes, very likely existed already in the most recent common ancestor of angiosperms and gymnosperms about 300 MYA, and *AGL2*-, *AGL17*-, and *SQUA*-like genes, existed at least already in the most recent common ancestor of monocots and eudicots about 200 MYA [5]. So some important events during the phylogeny of species, especially spermatophyte, can be shown through the evolution of MADS gene family [55]. And the soybean genome has experienced two WGD events, the legume WGD and Glycine WGD, after the Gamma WGT event, when the monocots and eudicots diverged [27], [28]. So the trace of all soybean MADS genes experienced the three rounds of the WGD events should been found in the soybean genome during the soybean evolution process. But some evidences were found only in the MIKC gene evolutions. The homologous blocks containing MADS genes showed that ancestors of 6 soybean MIKC^C^ subfamilies, SOC (TM3-like), SQUA, AGL6, SVP (STMADS11-like), AGL17, DEF/GLO, existed at least before the Gamma WGT event (Figure S5), and that *AG* and *SEP* (AGL2-like) at least before the legume WGD event (Figure 3). The evolution traces of blocks embodying 3 subfamilies of MIKC (AGL12, FLC and ABS), MIKC* family and all the type I MADS genes were only found after the *Glycine* WGD event (Figure 3 and 4 and Table S3). That indicated that in the soybean genome evolution, type I MADS-box genes and three MIKC subfamilies and MIKC* family had experienced faster birth-and-death evolution than some MIKC subfamilies.

### More Early Divergence of Paraog genes, More Significant Difference of Expressions

In the soybean, duplication events result in a genome with approximately 46,430 ‘high-confidence’ genes, of which 75% are present as more than one copy [28]. And approximately 50% of paralogs from the recent WGD event differentially expressed and thus had undergone expression sub-functionalization through RNA-seq expressions of 7 tissues [29]. Based on our results, most MIKC paralogs from the recent WGD event showed the similar expression profiles, and one of the paralog pair expressed more highly in the most tissue than the other (Figure 13). But for the paralogs from much deeper duplications, the detached transcriptions were more obvious. For example, three paralog gene pairs of AGL6 subfamily diverged into two clades after the Gamma WGT event, one clade experienced the two WGD events, and then formed one paralog gene pair, *GmMADS22/23*, and the other was composed of *GmMADS34/36* and *GmMADS21/91*(Figure 3). The former paralog gene pair strongly expressed in the flower and seeds (Figure 3 and 7), but the two latter paralog genes detached after the legume WGD event highly expressed in the roots at the flowering time (Figure 3 and 12). And other paralog genes of MIKC^C^ subfamilies from different divergence events show similar expression divergences to AGL6 subfamily. That indicated the Gamma WGT event had important roles in vaiable functions of paralogs.

### MADS Genes have Important Potential Roles in the Seed Development

Compared with type II *MADS*, very little is known about type I *MADS*, but recent studies display a key regulatory role for type I *MADS* factors in plant reproduction, in particular in specifying female gametophyte, embryo, and endosperm development [21], [48], [49], [56]–[58]. The rice and Arabidopsis have 28 and 61 type I *MADS* genes, respectively (Table S7). According to microarray expressions of *MADS* genes in rice or Arabidopsis (Figure S6 and S7), 10 and 32 type I *MADS* genes highly express in the rice or Arabidopsis seed development processes respectively. In addition, activities of promoters of 38 type I *MADS* genes are detected in the female gametophyte and seed development processes through their own promoters and 20 type I *MADS* genes are not detected [49]. In the soybean, 45 out of 75 type I *MADS* genes expressed in the seed tissues and transcriptions of 22 genes were not detected (Figure 4).

Since the first MADS genes are found in *Arabidopsis* and *Antirrhinum* respectively [3], [4], the importance of best-studied *MIKC^C^* genes is well known for floral homeotic functions during the ontogeny of flowers. Floral organ identity genes have been subdivided into five different classes, termed as class A (e.g. *AP2* and *AP1*), B (e.g. *PI*, *AP3*, *GLO*, *DEF*), C (e.g. *AG*), D (e.g. *SHP* and *STK*), and E (e.g. *SEP*) genes, which are required in different combinations to specify sepals, petals, stamens, carpels and ovules [10], [25], [59]–[61]. Based on *in silico* analysis and our RT-qPCR results, *GmMADS* genes displayed high transcriptions in the soybean flower. For example, 7 members of DEF/GLO subfamily highly expressed in the flowers (Figure 3 and 7). Transcriptions of all SQUA subfamily genes, especially the AP1 ortholog genes *GmMADS24*, *25*, *26*, and *27*, were also detected in the flowers (Figure 3 and 7).

But some MIKC^C^ subfamilies with known floral homeotic functions displayed high relative expression not only in the flowers, but in the seed tissues, especially in the early stage seeds (Figure 3 and 8). Among 11 members of SEP subfamily, 7 SEP-like genes (*GmMADS14*, *16*–*19*, *33*, and *71*) relatively highly expressed in the flowers, but all members of SEP subfamily strongly expressed in the seeds at different developmental stages (Figure 3, 6 and 8). In the rice, high transcriptions of four SEP genes (*OsMADS1*, *5*, *7/45*, *8/24*,) were detected both in the rice panicle and seed development and OsMADS34 were detected only in the rice panicle development (Figure S6). In Arabidopsis, *SEP1*, *2* and *3* expressed in embryonic culture tissues [48], and *SEP1* and *2* expressed both in the seed and in the flowers, while *SEP4* highly expressed in the flowers (Figure S7). That suggested that soybean SEP genes may play a fundamental role in the development of all floral organs and seeds.

Homeotic C-class gene *AG* ortholog genes, *GmMADS1* and *2* showed relatively high expression in the flowers and seeds (Figure 8), however, another *AG* ortholog gene, *GmMADS3* highly expressed not in the flowers but in the early seeds, as well as 2 *STK*-like genes (a paralogous gene pair, *GmMADS38*/*39*) and 4 *SHP2*-like genes (two paralogous gene pairs, *GmMADS11*/*13* and *GmMADS12*/*20*) did (Figure 6). And in the rice, compared with transcriptions in flowers, four rice AG members, such as *OsMADS3*, *13*, *21* and *66*, highly expressed in the seeds (Figure S6). In Arabidopsis, *STK* (*AGL11*) is not only detected in inflorescence but in the developing silique tissues, and redundantly with *SHP1*, *SHP2* and *ABS* regulate the seed development (Figure S7) [62]–[65]. The results indicated that *GmMADS* genes underwent different evelutional progress from that of Arabidopsis MADS gene did and acquired new function in seed development. Another *SHP1* ortholog gene *GmMADS74* were detected in the seeds and flowers at low level, but high expressed in the roots and leaves (Figure 12), inferring its function beyond flowers and seeds.

The embryo proper represents new sporophytic generation and contains the shoot and root meristems. It is well known of the *MIKC* genes, *AGL15* mRNA accumulates primarily in the embryo and the seed, and has an important component of the regulatory circuitry directing seed-specific processes in the developing embryo [62], [66], [67], and according to Figure S6, the expressions of *AGL15* and *AGL18* were increasing during the process of the seed mature. *GmAGL15* (*GmMADS122*) is also preferentially expressed in developing embryos, but not in the flowers and yang pods [68]. Both *AGL15* subfamily genes, *GmMADS122* and *43*, expressed in the seed tissues or flowers based on our RT-qPCR results, (Figure 6 and Table S6); two *AGL17* subfamily members *GmMADS64* and *160* highly accumulated in the globular stage embryo and low in the seed development (Table S6). In the rice, three AGL17-like genes, *OsMADS25*, *59* and *61*, highly expressed in the later stage of the seed development. But in Arabidopsis, three AGL17-like genes, *AGL17*, *21* and *ANR*, highly expressed only in the roots, and low expressions of *AGL16* can be detected in seeds (Figure S7). The suspensor is a terminally differentiated structure that supports and nourishes the embryo proper and degenerates later in development. *GmMADS51* and *52* (*SVP*), *GmMADS106* (*SOC*) and *GmMADS127* (*AGL17*) highly expressed in suspensors and had potential roles in the function of the suspensors.

## Materials and Methods

### Plant Materials

The soybean (*Glycine max*) cultivar Kennong 18 was employed in all experiments. Plants were grown in a growth chamber under short day conditions (8 hr light/16 hr dark) at a temperature 25°C ∼ 28°C. Under the normal conditions, tissues were separately harvested at different stages for gene expression analysis. The seeds were sampled at day 7, 14 and 21 after flowering and when the seeds became yellow. At least five individual plants per sample were then harvested and frozen in liquid nitrogen and stored at −80°C until used. And all experiments were repeated three times under the consistent conditions.

### Identification, Classification and Motif of Soybean MADS Genes

According to the HMM model of SRF-type MADS transcription factor (PF00319), HMMER v3.0 [69] were employed to identify 163 soybean MADS genes through searching the soybean genome protein database (V1.01, http://www.phytozome.net/soybean.php). The fragment genes were predicted the whole CDS by FGENESH (http://linux1.softberry.com/berry.phtml) or blasting the NCBI Ref-RNA database (Table S1). And 163 candidate genes were retrieved and named as *GmMADS1* through *163* (Table S1).

*V. vinifera* and *M.truncatula* V3.5 whole-genome protein sequences were retrieved from Phytozome v8.0 (http://www.phytozome.net/) and the website (http://www.medicagohapmap.org) respectively, to investigate MADS inter-species colinearity. And their MADS families were also screened through HMMER v3.0 (Table S7). Some of 54 *V.vinifera* MADS family were named according to Diaz-Riquelme, *et al*. [42] and other named as *VvMADS55* through *78*, and *M. truncatula* MADS family were named as *MtMADS1* through *93*.

Because diversity of MADS genes in *Arabidopsis* is rather ancient and representative for other flowering plants [5], 108 Arabidopsis [14] and 75 rice MADS genes [41] (Table S7) were selected to classify 163 soybean MADS proteins into 5 families, MIKC^C^, MIKC*, Mα, Mβ and Mγ, and soybean genes most similar to Arabidopsis MADS genes were considered as the *Arabidopsis* ortholog genes, as well as the classification of *M. truncatula* and *V.vinifera* MADS family. And a topology tree (Figure S1) was constructed to investigate the relationship of the soybean and Arabidopsis MADS proteins through ClustalW1.8 and MAGE 5.0 using Neighbor-Joining method [70].

To analyze the specific motifs of different MADS families, 10 motifs were identified by MEME v 4.9.0 with default parameters [71] (http://meme.nbcr.net/meme/cgi-bin/meme.cgi) among the soybean, rice, *Arabidopsis*, *V.vinifera* and *M. truncatula* after removing redundant sequences by Purge tool [72], and then MAST v 4.9.0 was used to identify motif organizations of 163 soybean MADS proteins (Figure S2).

### Investigating QTLs Relative to Soybean MADS Genes

To determine the co-localization of MADS genes with the QTLs, available SSR or RFLP markers linked to the QTLs were downloaded from Soybase (http://www.soybase.org/dlpages/index.php) [46], and markers’ sequences were mapped to the soybean genome (v1.09) through Blast. Furthermore, the QTL physical locations are usually uncertain due to the recombination frequency being affected by population size, even if the linked marker sequences and their genomic positions are known. Therefore, genes within a 2-Mb genomic region flanking markers were associated with a QTL.

### Total RNA Isolation and Quantitative Reverse Transcription-PCR

The procedure used for RNA extraction, cDNA synthesis, and PCR was as described by Hu, et al [73]. According to the specificity and efficiency of the primer pairs, 96 soybean MADS genes were designed by Beacon Designer 7.9, and at least one primer was specific for the target gene primer pairs (Table S1). *GmSKIP* (Glyma12g05510), *GmUKNII* (Glyma14g08990) and *GmUKN1* (Glyma12g02310) were selected as reference genes for all the experiments [73]. And primers used as controls or for analysis had an efficiency of greater than 90% by LinRegPCR (http://LinRegPCR.HFRC.nl) [74].

### *In silico* Expression Analysis of MADS Genes

The tissue-specific transcript characteristics of 163 MADS genes were investigated based on the RNA-seq data from 17 soybean tissues (http://seedgenenetwork.net/soybean), such as three seed compartments at the seed globular stage (GSE29162), whole seeds at five stages of seed development (globular, heart, cotyledon, early-maturation, dry), and vegetative (leaves, roots, stems, seedlings) and reproductive (floral buds) tissues (GSE29163), cotyledons of mid-maturation and late maturation seeds, whole dry seeds, and cotyledons of seedlings six days after imbibitions (GSE29134).

For the whole expression analyses of soybean MADS, the RPKM method was employed to correct for biases in total gene exon size and to normalize for the total short read sequences obtained in each tissue library [53], [54]. And the geometrical average of RPKM values of the selected reference genes in RT-qPCR experiments was a reference gene value, and the ratios of target gene and a reference gene value were the relative expression level of the target gene in each sample (Table S6). A hierarchical clustering analysis of gene-wide normalizations of 138 gene transcription profiles in 17 tissues using a Pearson correlation was computed by Gensis1.7.5 [75].

Compared with expression profiles of the soybean MADS in the seed development, the microarray data, GSE6893 for rice [76] and GSE680 for Arabidopsis [77], were downloaded from the GEO database, and then cDNAs of *MADS* genes were selected to identify special probe sets through Probe Match tool (http://www.affymetrix.com/analysis/index.affx). At last, expression values of 55 rice and 73 Arabidopsis *MADS* probe sets were computed by Genespring 11.5 respectively (Figure S6 and 7). A hierarchical clustering analysis of gene-wide normalizations of transcription profiles used a Pearson correlation by Gensis1.7.5 [75].

### Collinear Relationships of 163 MADS Genes in the Soybean Genome

MCScanX [52] was employed to identify syntenic regions containing MADS genes among soybean (Table S3), *V. vinifera* and *M. truncatula*. Briefly, BLASTP with e-value ≤1e−10 was applied to find intra-species paralogous pairs and inter-species homologous pairs, and the homologous blocks involved at least 5 collinear gene pairs and the gap gene pairs number was not more than 20. Based on the average *Ks* value of homologous blocks, the divergence times of the blocks were computed to investigate the evolution of soybean MADS genes in the soybean genome evolution. For example, if the average *Ks* is less than 0.3, divergence of the homologous blocks is about after the *Glycine* WGD event, and *Ks* is more than 1.5, the divergence time is after the Gamma WGT event, and *Ks* is between 0.3 and 1.5, the divergence time is after the Legume WGD event and before the *Glycine* WGD event.

Besides the WGD duplication, Soytedb (http://www.soybase.org/soytedb/) [35] was employed to identified the nearest transposable elements around some soybean MADS genes to analyze the importance of the transposable duplications to the soybean MADS gene evolutions (Table S4).

## Supporting Information

Figure S1 **Phylogenetic relationship of MADS genes between** ***Glycine max*** **and** ***Arabidopsis*** **.** The deduced full-length amino acid sequences of 163 *Glycine* and 108 *Arabidopsis* genes were aligned by Clustal X 1.83 and the phylogenetic tree was constructed using MEGA 5.0 by the Neighbor-Joining (NJ) method with 1,000 bootstrap replicates. Lines of each *GmMADS* subfamily are in a specific color or in different color background.
(TIF)

Figure S2 **Motifs of 163 soybean MADS proteins.** Motif 1 and 2 are equivalent to the MADS-box domain (PF00319), and motif 3 and motif 6 are equivalent to the part of the I-domain and K-box domain (PF01486) for type II MADS proteins, respectively. Other motifs was unknown. Ten motifs were identified through MEME (http://meme.nbcr.net/meme/), and then motif organizations of 163 soybean MADS were investigated through MAST (http://meme.nbcr.net/meme/).
(TIF)

Figure S3 **Expression heatmap of 138 MADS genes in the 17 tissues through RNA-seq.** A hierarchical clustering analysis of gene-wide normalizations of 138 gene transcription profiles in 17 tissues using a Pearson correlation by Gensis1.7.5 suggested 138 genes can be grouped into 13 expression clusters. Clusters in same color showed the genes expressed mainly in the same tissues. And other notes as Figure 3.
(TIF)

Figure S4 **Expression heatmap of 82 MADS genes in the 12 tiessues through RT-qPCR.** The lines in same colors showed the genes expressed mainly in the same tissues. A hierarchical clustering analysis of gene-wide normalizations using a Pearson correlation by Gensis1.7.5 suggested 82 genes can be grouped into 5 clusters. And other notes as Figure 6.
(TIF)

Figure S5 **The evolution model of blocks embodying MADS genes in the soybean.** The black blocks were as the ancestors before the Gamma WGT event, and red, blue and green blocks showed the traces of the Gamma WGT, Legume WGD and Glycine WGD event respectively, and the bars were the MADS genes in the blocks. And the blocks without color showed the blocks were lost in the genome evolutionary history. Green blocks without bar were that the MADS genes were lost after the WGD events.
(TIF)

Figure S6 **Microarray expressions of rice** ***MADS*** **genes.** The microarray data (GSE6893) were from the NCBI GEO database. Special probe sets for 55 rice *MADS* genes and expression values were computed through Genespring 11.5. A hierarchical clustering analysis of gene-wide normalizations of 55 gene transcription profiles in 14 tissues using a Pearson correlation by Gensis1.7.5. SAM, P1 to P6 were up to 0.5 mm, 0–3 cm, 3–5 cm, 5–10 cm, 10–15 cm, 15–22 cm and 22–30 cm of panicles, respectively. And S1, S2, S3, S4 and S5 were seeds at 0–2, 3–4, 5–10, 11–20 and 21–29 days after pollination, respectively. Root and Young leaves was the roots and leaves from 7-d-old seedlings, respectively. Young leaves were as the control.
(TIF)

Figure S7 **Microarray expressions of Arabidopsis** ***MADS*** **genes.** The microarray data (GSE680) were from the NCBI GEO database. Special probe sets for 73 rice *MADS* genes and expression values were computed through Genespring 11.5. A hierarchical clustering analysis of gene-wide normalizations of 73 gene transcription profiles in 11 tissues using a Pearson correlation by Gensis1.7.5. Seedling was as the control.
(TIF)

Table S1 **The information of 163** ***GmMADS*** **genes.**
(XLSX)

Table S2 **The best match sequence of the soybean MADS motif.**
(XLSX)

Table S3 **Intra- or inter-species synteny of soybean MADS.**
(XLSX)

Table S4 **Proximal transposable elements of some soybean MADS genes.**
(XLSX)

Table S5 **QTLs relative to soybean** ***MADS*** **genes.**
(XLSX)

Table S6 **The normaliztion transcriptions of soybean MADS genes through RNA-seq analysis.**
(XLSX)

Table S7 **The information of MADS family in** ***A. thaliana*** **,** ***M. truncatula*** **and** ***V. vinifera***.
(XLSX)
