# Supplementary figures and images for: Genome-Wide Expression Analysis of Soybean MADS Genes Showing Potential Function in the Seed Development
Source: PLoS One. 2013 Apr 30;8(4):e62288. doi: 10.1371/journal.pone.0062288 (PMC3640087; doi:10.1371/journal.pone.0062288)

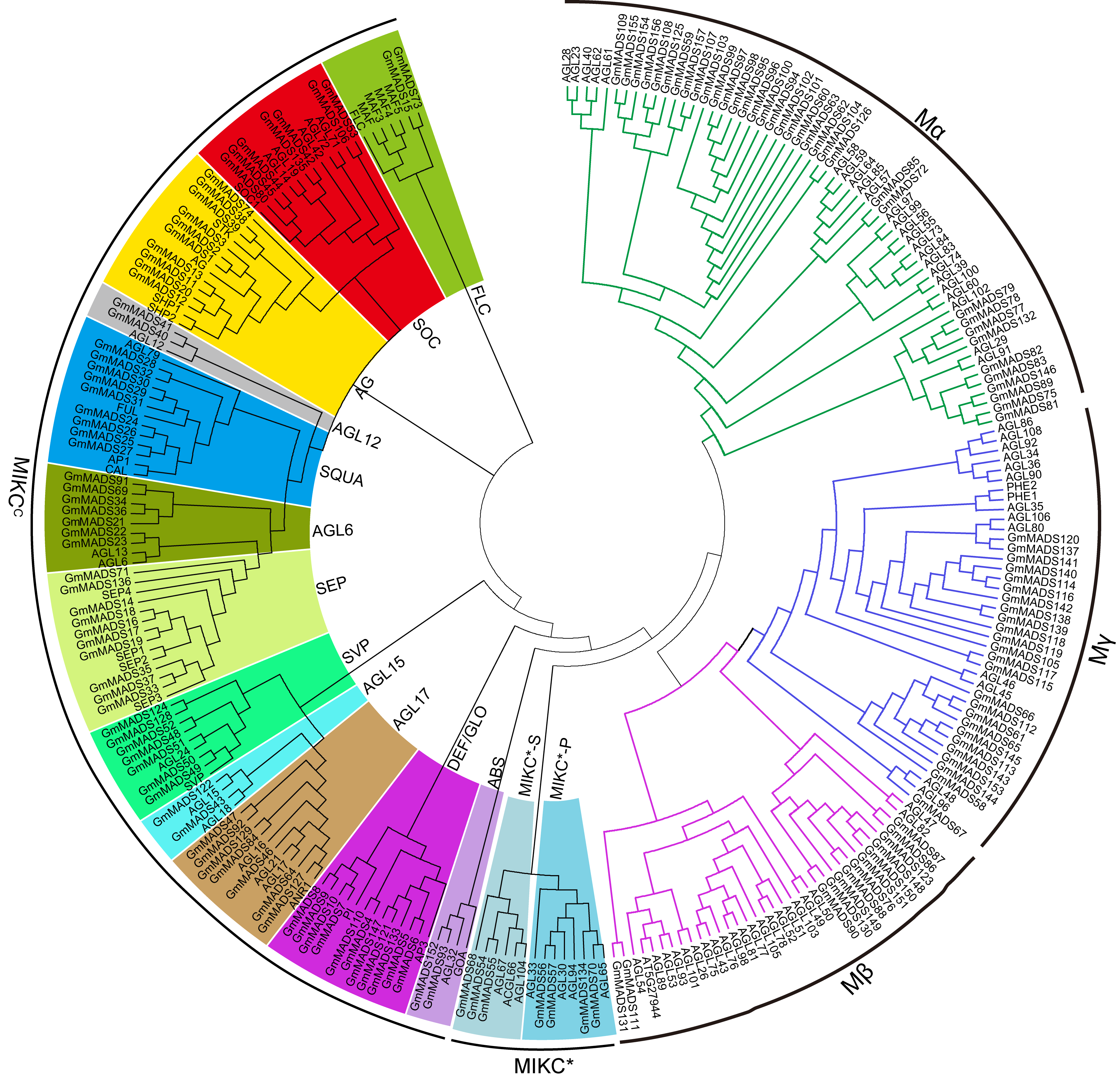

Supplement: Figure S1 — Phylogenetic relationship of MADS genes between Glycine max and Arabidopsis . The deduced full-length amino acid sequences of 163 Glycine and 108 Arabidopsis genes were aligned by Clustal X 1.83 and the phylogenetic tree was constructed using MEGA 5.0 by the Neighbor-Joining (NJ) method with 1,000 bootstrap replicates. Lines of each GmMADS subfamily are in a specific color or in different color background. (TIF) [file pone.0062288.s001.tif]

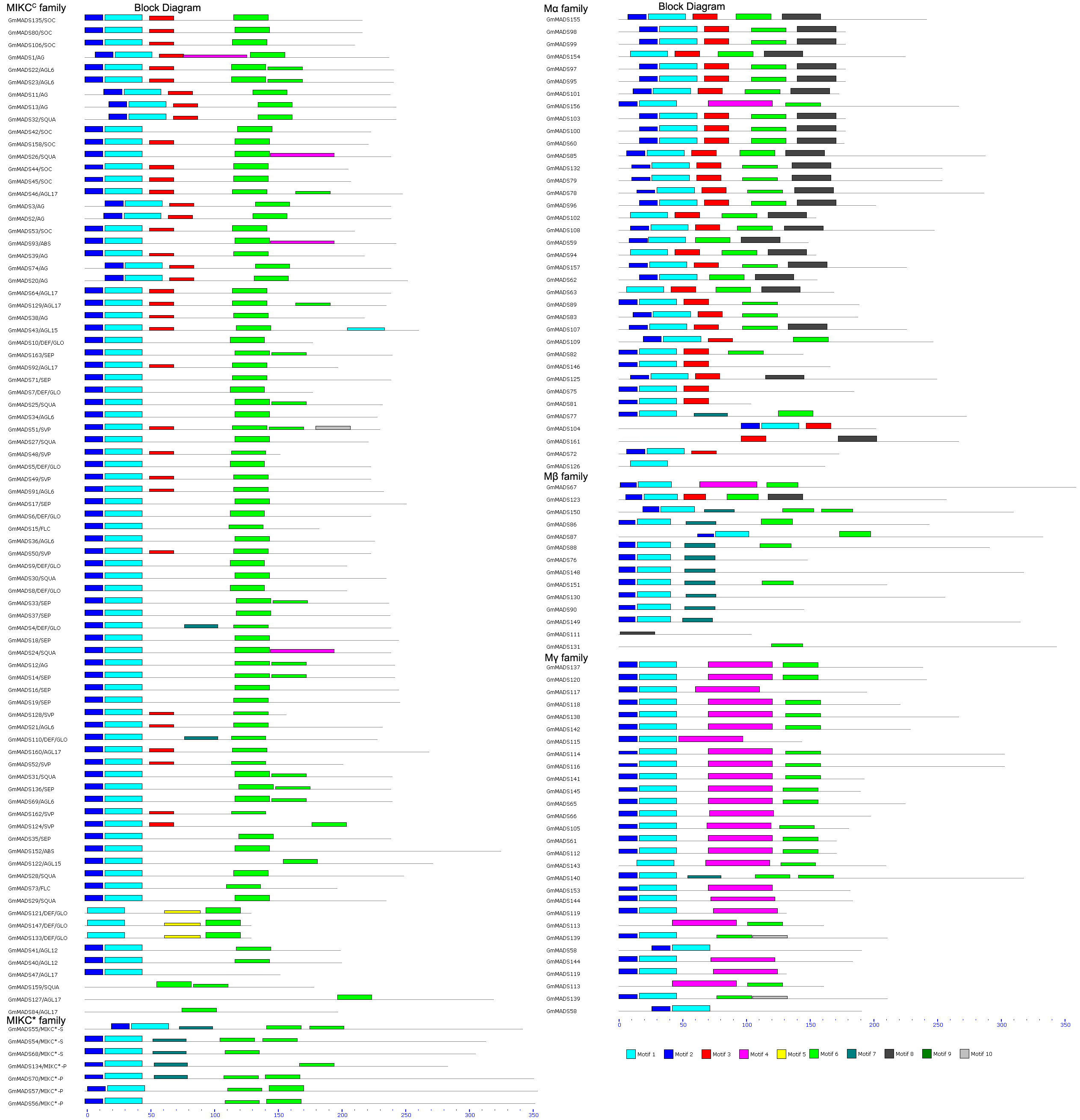

Supplement: Figure S2 — Motifs of 163 soybean MADS proteins. Motif 1 and 2 are equivalent to the MADS-box domain (PF00319), and motif 3 and motif 6 are equivalent to the part of the I-domain and K-box domain (PF01486) for type II MADS proteins, respectively. Other motifs was unknown. Ten motifs were identified through MEME (http://meme.nbcr.net/meme/), and then motif organizations of 163 soybean MADS were investigated through MAST (http://meme.nbcr.net/meme/). (TIF) [file pone.0062288.s002.tif]

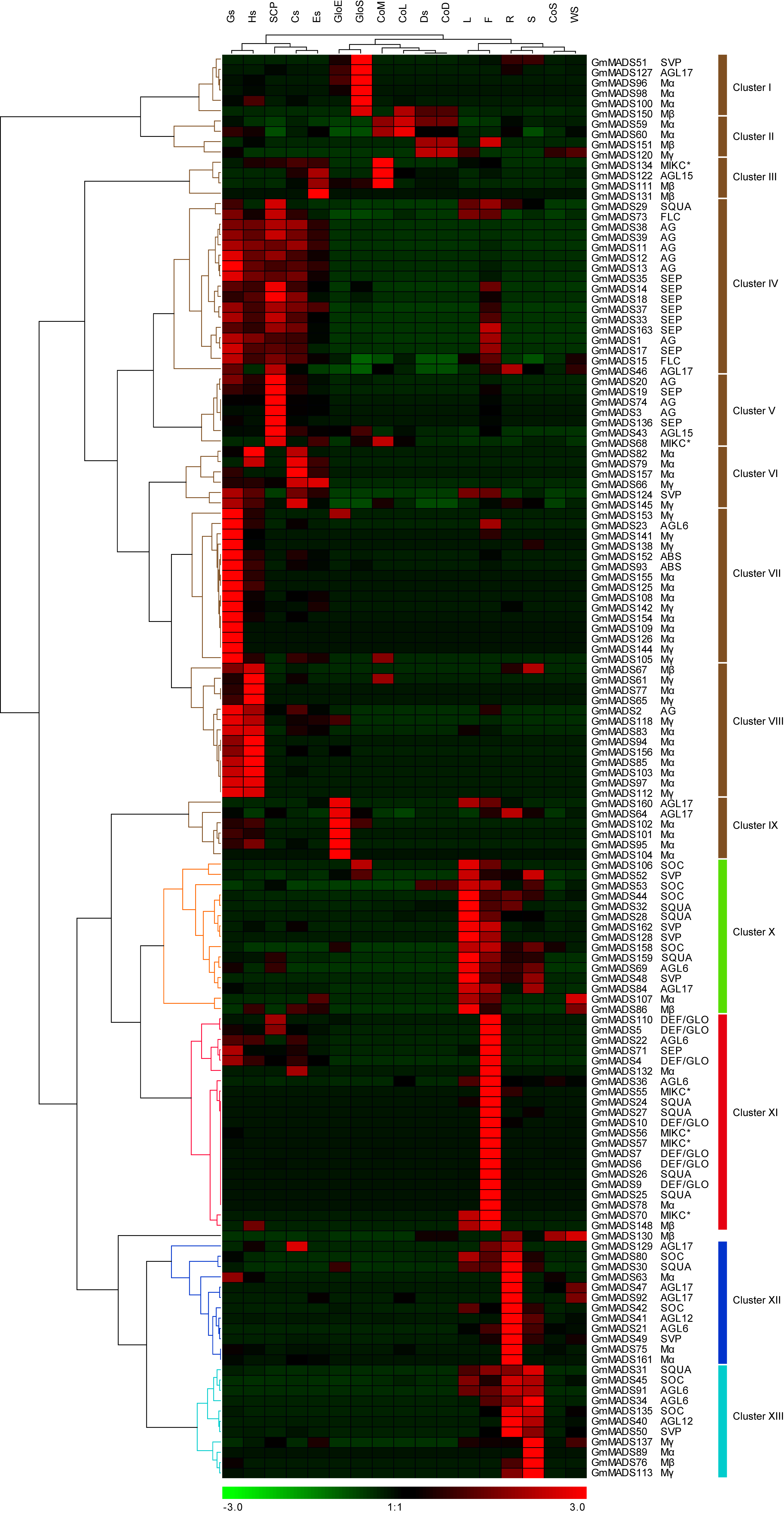

Supplement: Figure S3 — Expression heatmap of 138 MADS genes in the 17 tissues through RNA-seq. A hierarchical clustering analysis of gene-wide normalizations of 138 gene transcription profiles in 17 tissues using a Pearson correlation by Gensis1.7.5 suggested 138 genes can be grouped into 13 expression clusters. Clusters in same color showed the genes expressed mainly in the same tissues. And other notes as Figure 3. (TIF) [file pone.0062288.s003.tif]

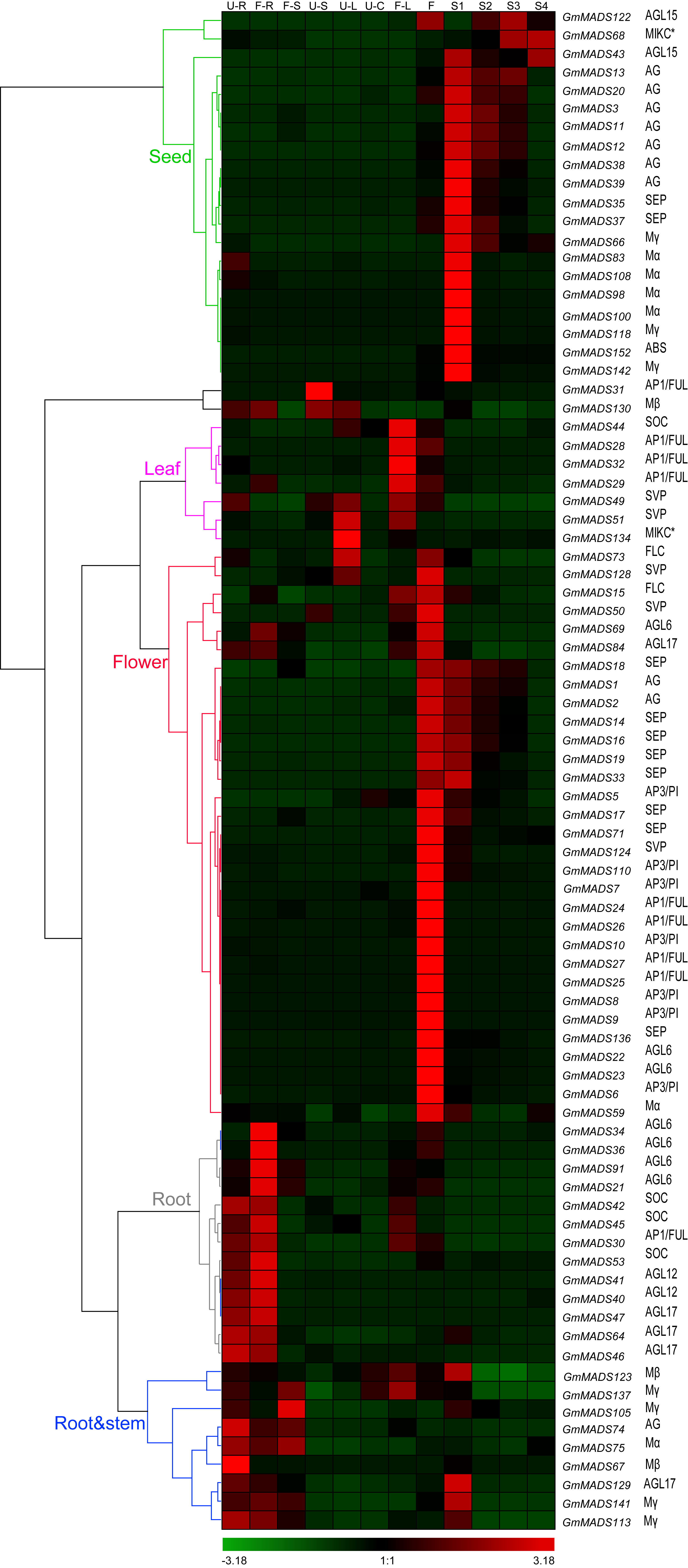

Supplement: Figure S4 — Expression heatmap of 82 MADS genes in the 12 tiessues through RT-qPCR. The lines in same colors showed the genes expressed mainly in the same tissues. A hierarchical clustering analysis of gene-wide normalizations using a Pearson correlation by Gensis1.7.5 suggested 82 genes can be grouped into 5 clusters. And other notes as Figure 6. (TIF) [file pone.0062288.s004.tif]

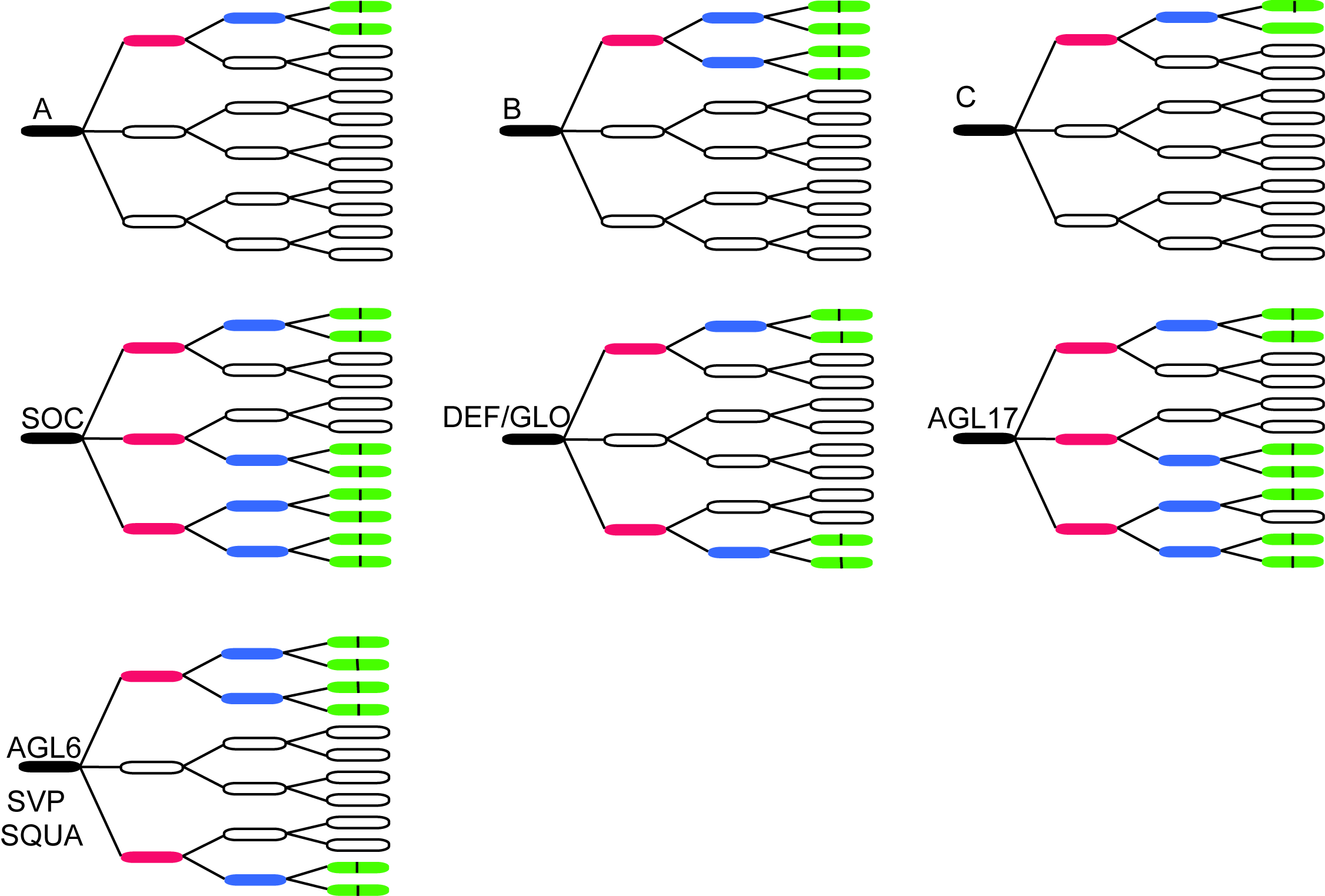

Supplement: Figure S5 — The evolution model of blocks embodying MADS genes in the soybean. The black blocks were as the ancestors before the Gamma WGT event, and red, blue and green blocks showed the traces of the Gamma WGT, Legume WGD and Glycine WGD event respectively, and the bars were the MADS genes in the blocks. And the blocks without color showed the blocks were lost in the genome evolutionary history. Green blocks without bar were that the MADS genes were lost after the WGD events. (TIF) [file pone.0062288.s005.tif]

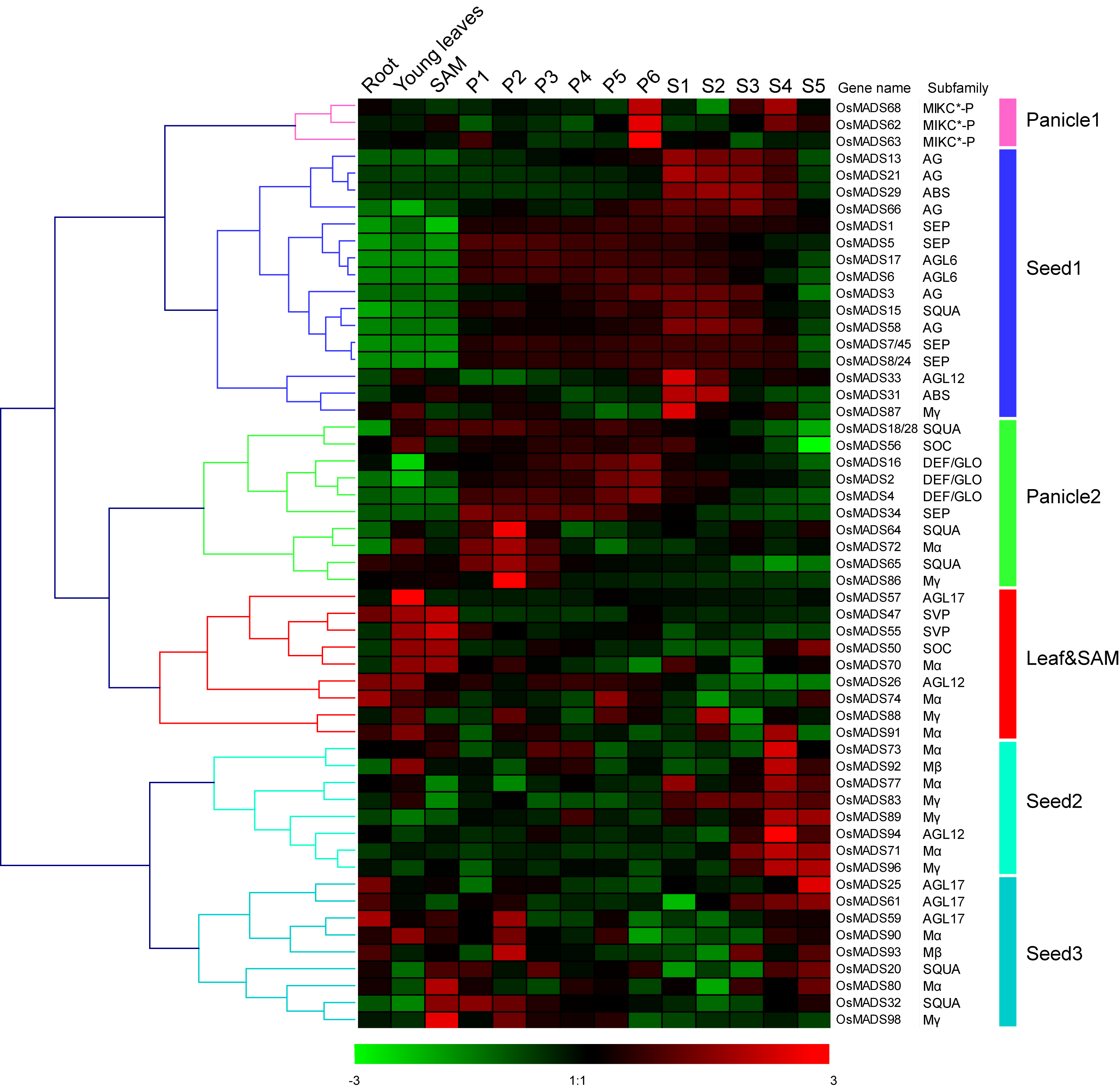

Supplement: Figure S6 — Microarray expressions of rice MADS genes. The microarray data (GSE6893) were from the NCBI GEO database. Special probe sets for 55 rice MADS genes and expression values were computed through Genespring 11.5. A hierarchical clustering analysis of gene-wide normalizations of 55 gene transcription profiles in 14 tissues using a Pearson correlation by Gensis1.7.5. SAM, P1 to P6 were up to 0.5 mm, 0–3 cm, 3–5 cm, 5–10 cm, 10–15 cm, 15–22 cm and 22–30 cm of panicles, respectively. And S1, S2, S3, S4 and S5 were seeds at 0–2, 3–4, 5–10, 11–20 and 21–29 days after pollination, respectively. Root and Young leaves was the roots and leaves from 7-d-old seedlings, respectively. Young leaves were as the control. (TIF) [file pone.0062288.s006.tif]

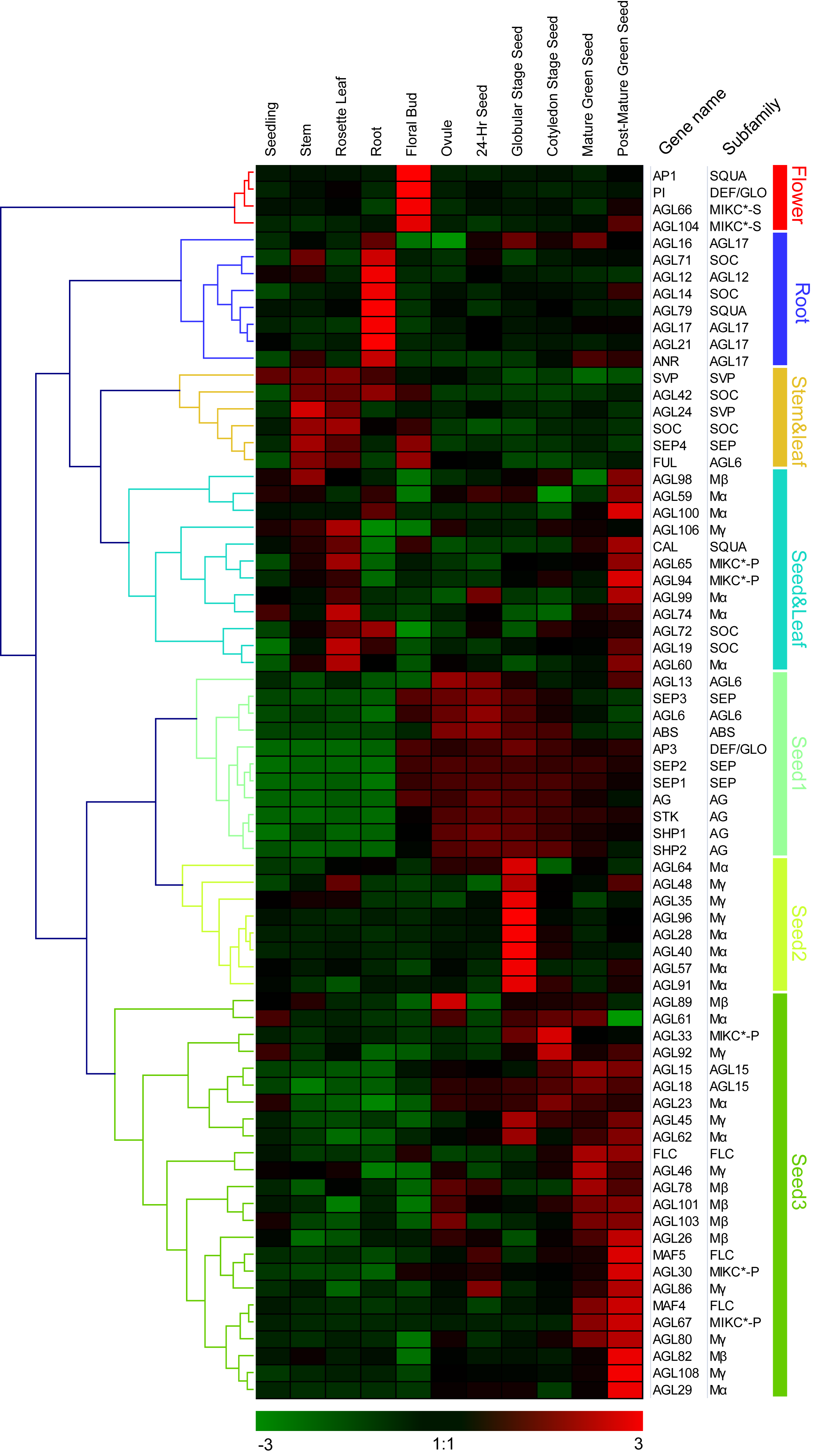

Supplement: Figure S7 — Microarray expressions of Arabidopsis MADS genes. The microarray data (GSE680) were from the NCBI GEO database. Special probe sets for 73 rice MADS genes and expression values were computed through Genespring 11.5. A hierarchical clustering analysis of gene-wide normalizations of 73 gene transcription profiles in 11 tissues using a Pearson correlation by Gensis1.7.5. Seedling was as the control. (TIF) [file pone.0062288.s007.tif]
